# Antiretroviral Therapy in Prevention of HIV and TB: Update on Current Research Efforts

**DOI:** 10.2174/157016211798038597

**Published:** 2011-09

**Authors:** Reuben Granich, Somya Gupta, Amitabh B Sutha, Caoimhe Smyth, David Hoos, Marco Vitoria, Mariangela Simao, Catherine Hankins, Bernard Schwartlander, Renee Ridzon, Brigitte Bazin, Brian Williams, Ying-Ru Lo, Craig McClure, Julio Montaner, Gottfried Hirnschall

**Affiliations:** 1 World Health Organization, Geneva, Switzerland;; 2 UNAIDS, Geneva, Switzerland;; 3 Bill and Melinda Gates Foundation, Seattle, United States of America;; 4 National Agency for Research on AIDS and Viral Hepatitis (ANRS), Paris, France;; 5 South African Centre for Epidemiological Modelling and Analysis, Stellenbosch, South Africa;; 6 British Columbia Centre for Excellence in HIV/AIDS, Vancouver, Canada

**Keywords:** HAART, highly active antiretroviral therapy, HIV prevention, randomised controlled trials, research activities, tuberculosis prevention.

## Abstract

There is considerable scientific evidence supporting the use of antiretroviral therapy (ART) in prevention of human immunodeficiency virus (HIV) and tuberculosis (TB) infections. The complex nature of the HIV and TB prevention responses, resource constraints, remaining questions about cost and feasibility, and the need to use a solid evidence base to make policy decisions, and the implementation challenges to translating trial data to operational settings require a well-organised and coordinated response to research in this area. To this end, we aimed to catalogue the ongoing and planned research activities that evaluate the impact of ART plus other interventions on HIV- and/or TB-related morbidity, mortality, risk behaviour, HIV incidence and transmission. Using a limited search methodology, 50 projects were identified examining ART as prevention, representing 5 regions and 52 countries with a global distribution. There are 24 randomised controlled clinical trials with at least 12 large randomised individual or community cluster trials in resource-constrained settings that are in the planning or early implementation stages. There is considerable heterogeneity between studies in terms of methodology, interventions and geographical location. While the identified studies will undoubtedly advance our understanding of the efficacy and effectiveness of ART for prevention, some key questions may remain unanswered or only partially answered. The large number and wide variety of research projects emphasise the importance of this research issue and clearly demonstrate the potential for synergies, partnerships and coordination across funding agencies.

## INTRODUCTION

Antiretroviral therapy (ART) reduces mortality and morbidity related to human immunodeficiency virus (HIV) infection. The need to provide expanded access to ART is now widely accepted, and there is a pressing demand for both increased investment and more efficient use of funding in order to achieve and sustain universal access [1]. Furthermore, the World Health Organization (WHO) estimates that less than 40% of people living with HIV know their status and there is a need for expanded access to HIV testing and counseling [2] both as a prevention measure itself and also as a gateway to other HIV services including care and treatment. Although ART has considerable potential to save lives while reducing HIV transmission [3-6], without a reduction in HIV incidence it is unlikely that we will be able to meet and sustain Universal Access targets by 2015 [2]. Indeed, in 2010 an estimated 58% of those eligible were not receiving ART. Besides individual benefits, ART has substantial potential to enhance prevention efforts because it suppresses HIV viral load, and therefore infectiousness, of persons already infected with HIV [5,7,8]. As a result, there is increasing scientific evidence supporting the use of ART in prevention of HIV and tuberculosis (TB) as part of broader combination prevention efforts [5,6,8]. The *HIV Prevention Trials Network (HPTN) 052* randomized controlled trial assessing the effect of early ART initiation on HIV incidence reduction in discordant couples announced their results early when the data and safety monitoring board found a 96% reduction in HIV transmission in the arm that received ART immediately between 550-350 CD4 cells/ mm^3^ versus deferral of ART to < 250 cells/mm^3^ [9]. While the results from HPTN 052 include a significant benefit for earlier ART for reducing extrapulmonary TB, there was no benefit for pulmonary TB and the results have raised questions regarding the overall value of earlier ART for prevention of TB. Observational studies suggest that ART has been associated with up to a 92% reduction in the incidence of tuberculosis, benefiting both people living with HIV and potentially reducing TB transmission to others [10,11]. Recognising these benefits, research is increasingly focusing on answering open questions regarding feasibility and cost-related issues of integrating ART into combination prevention approaches.

The current scale of HIV prevention efforts may reduce new infections but are unlikely to achieve sustained and widespread reduction in HIV incidence, and there have been widespread calls for intensification of prevention efforts in both scale and scope [12]. Interest in and exploration of ART in prevention has increased and there have been a number of stakeholders who have contributed to building understanding and the scientific evidence base [5,6]. New public health interventions frequently require convincing evidence and considerable time before they are implemented. For example, research on male circumcision took approximately 20 years and culminated in three definitive randomised controlled trials (RCTs) showing consistent efficacy [13-15]. Still a number of high HIV-burden countries have not adopted circumcision as part of HIV prevention policy [16]. Similarly, prevention of mother-to-child transmission of HIV took years of basic science and field research before RCTs supported the use of ART to prevent transmission [17-21]. Ongoing areas of prevention research have been recently reviewed and include behavioural and biomedical approaches including HIV testing and counselling (including couples testing and counselling); ART; oral and topical microbicides; pre-exposure prophylaxis; vaccines; and how best to package together these interventions for specific populations (“combination HIV prevention”) [22-26]. Additionally, medication-assisted therapy (MAT) for the treatment of co-morbid opioid and other drug and alcohol use disorders among HIV-infected persons is another form of prevention *via *preventing relapse to drug use thereby improving adherence to ART and decreasing HIV RNA levels [27-30]. Although biomedical interventions are more amenable to individual observational and randomised controlled trials, effective prevention strategies are rarely implemented in isolation and WHO recommends a combined approach [26, 31]. The complex nature of the HIV prevention response, resource constraints, remaining questions about cost and feasibility, and the need to use a solid evidence base to make policy decisions, and the implementation challenges to translating trial data to operational settings require a well-organised and coordinated response to research on ART in prevention of HIV and TB.

In November of 2009, WHO convened a meeting of stakeholders including researchers, HIV program managers, civil society representatives, people living with HIV, human rights experts and ethicists, donors and bilateral agencies on the topic of ART for prevention. The meeting included over 100 experts who reviewed the evidence base for ART in prevention of HIV and TB, discussed ethical issues, and examined broad issues around the concept of ART for HIV prevention, including feasibility and acceptability, human rights and ethical implications, and research priorities [32]. In May 2011, the British Columbia Centre for Excellence in HIV/AIDS (BCCfE) with co-organizers WHO, the Joint United Nations Programme on HIV/AIDS (UNAIDS), the International AIDS Society, the United States (US) National Institutes of Health (NIH) and other stakeholders, hosted a meeting that focused on ART in prevention research [33]. It focused on critically reviewing research related to the secondary preventive benefit (as it relates to HIV and TB transmission) of expanding ART coverage among people living with HIV, commonly referred as “Treatment as Prevention” (TasP). Participants also discussed research priorities including a number of ongoing and planned research projects. To assist with strategic planning and future policy formulation, this article identifies and reviews selected “ART in prevention of HIV and TB” research efforts.

## METHODS

We sought studies that evaluate the impact of ART plus other interventions on the HIV- and/or TB-related morbidity and mortality, risk behaviour, HIV incidence and transmission. We conducted a search on the websites of the National Library of Medicine, PubMed, NIH, HIV Prevention Trial Network (HPTN) and Clinical Trials.gov website to identify ongoing/planned research work on HIV and ART. The search strategy included the keywords “HIV and ART”, with “treatment”, “prevention”, “research”, "campaign" and “tuberculosis” alone and in combination. Out of nearly 500 studies, selected studies pertaining to evaluating the impact of ART plus other interventions on HIV- and/or TB-related morbidity and mortality, risk behaviour, HIV incidence and transmission were obtained. Community-based studies, randomised controlled trials, cohort studies and other types of studies were included. Scientific experts in this area of research, including funding agencies were contacted for information on current projects. They were asked to review the list of research projects on ART treatment in the prevention of HIV and TB to determine if the list was comprehensive and ensure all projects were relevant to the topic. The bibliographies of studies selected to be included in this article were again searched for additional references. We focused on explicit ART for prevention research studies and excluded studies that focused on other aspects of expanding ART or TB treatment (e.g. adherence, best regimens,) and published studies (e.g. *HPTN 052*). The following project details were extracted and summarized: focus of research, study design, principal interventions, primary outcome assessed, region, time period, agency and sponsors (if funded).

## RESULTS

### Summary of Studies

**Fig. (1):**
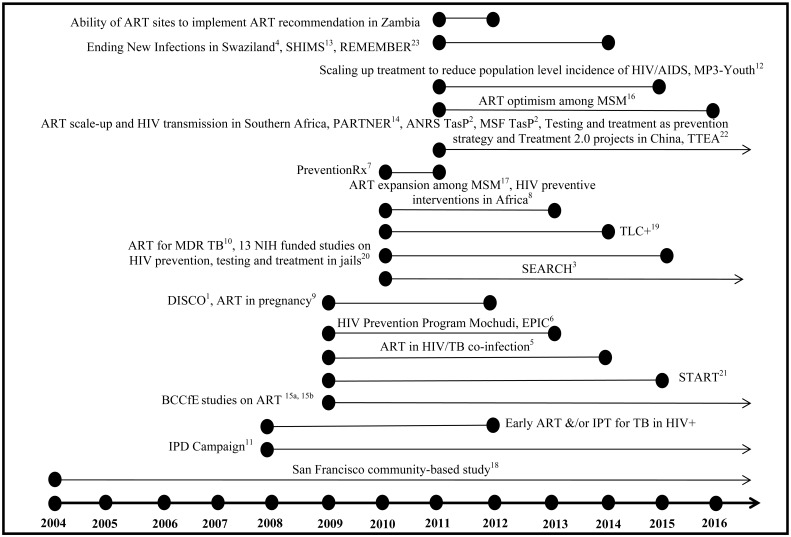
Timeline of projects on antiretroviral therapy (ART) in prevention of HIV and tuberculosis (TB). Note: Figure includes studies with an available timeline—others not listed.
DISCO - Discordant Couples Cohort TeamTasP – Treatment as PreventionSEARCH - Sustainable East Africa Research for Community HealthMaxART Maximizing ART for Better Health and Zero New InfectionsImmunology and Outcomes after HAART in HIV/TB co-infectionEPIC – Enhance Prevention in CouplesMulti-component, Targeted HIV Prevention for Sub-Saharan Africa: PreventionRxInterventions to Decrease HIV Infectiousness in Uganda and South AfricaHIV/HAART and Pregnancy/Contraception in Rakai, UgandaImpact of HIV, ART and TB Genotype on Survival in MDR TBIPD Campaign – Integrated Prevention Demonstration CampaignMP3-Youth - Gender-specific Combination HIV Prevention for Youth in High-Burden SettingsSHIMS - Swaziland HIV Incidence Measurement SurveyPARTNER - Partners of people on ART: a New Evaluation of the RisksEffect of early versus deferred ART for HIV on survival.Association of highly active antiretroviral therapy coverage, population viral load, and yearly new HIV diagnoses in British Columbia, Canada: a population-based study
HAART Optimism, Drug Use and Risky Sexual Behaviour among MSM in British ColumbiaEffect of HAART Expansion on Community Levels of HIV Viral Load and HIV Risk Behaviours among MSM in British ColumbiaDecreases in Community Viral Load Are Accompanied by Reductions in New HIV Infections in San FranciscoHPTN-065, TLC+: A Study to Evaluate the Feasibility of an Enhanced Test, Link to Care, Plus Treat Approach for HIV Prevention in the United States13 NIH (National Institutes of Health, USA) funded studies on HIV prevention, testing and treatment in jails
A Randomized Controlled Trial and Cohort Study of HIV Testing and Linkage to CareEffectiveness of Peer Navigation to Link Released HIV+ Jail Inmates to HIV CareImproving Linkage to HIV Care Following Release from IncarcerationRandomized Control Trial of an augmented test, treat, link, & retain model for North Carolina and Texas PrisonersSeek, Test, Treat: An Integrated Jail-Prison-Community Model for IllinoisSeek, Test, and Treat StrategiesCARE Corrections: Technology for Jail HIV/HCV Testing, Linkage, and Care (TLC)Finding, Testing and Treating High-risk Probationers and Parolees with HIVSTART Together: HIV Testing and Treatment in and after JailSeek, Test, Treat Strategies for Vietnamese Drug Users: A Random Controlled TrialAlcohol Pharmacotherapies among Released HIV+ PrisonersNaltrexone for Opioid Dependent Released Human Immunodeficiency Virus Positive (HIV+) Criminal Justice PopulationsHIV, Buprenorphine, and the Criminal Justice System
START - Strategic Timing of Antiretroviral TreatmentTTEA - Test and Treat to End AIDSREMEMBER - The Reducing Early Mortality and Early Morbidity by Empiric Tuberculosis Treatment Regimens study

**Fig. (2):**
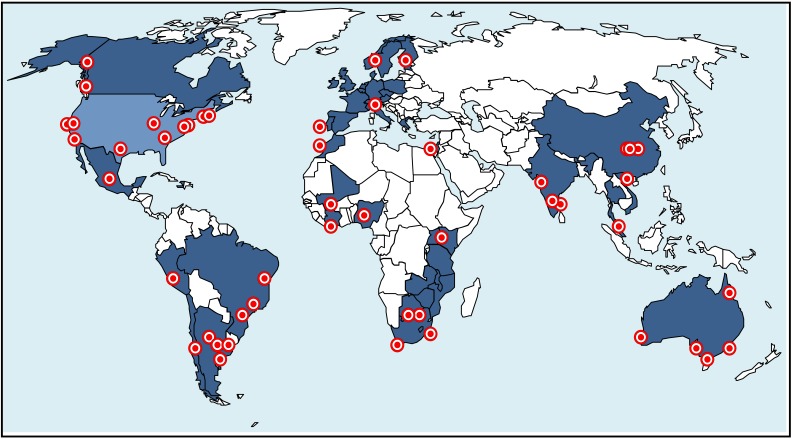
Map representing countries planning or implementing antiretroviral therapy (ART) in prevention of HIV and/or tuberculosis (TB) research. Dark blue represents countries that are conducting ART in prevention of HIV and/or TB research, light blue represents countrywide efforts (United States, Swaziland), and the red dots represent selected study sites within countries conducting research (some countries had too many sites to represent on this graphic).

Our initial search yielded nearly 500 studies, of which 50 were taken forward for full review. Of the 50, 20 were from North America, 22 from Africa, 4 from Asia, 1 from Europe and 3 were multisite international studies. Of the 24 randomised control trials (individual or community cluster), 12 of the planned or ongoing studies were from resource-constrained settings. (See Table **1** and Figs. **1**, **2**).

### Region: Africa

*Scaling Up Treatment to Reduce Population Level Incidence of HIV/AIDS* is a four-year study designed by investigators at the British Columbia Centre for Excellence in HIV/AIDS in British Columbia, Canada along with the Joint Clinical Research Centre in Uganda. This study uses a randomised step-wedge design to examine the impact of increased access to ART in Ugandan regions on incidence measured by home-based testing. Because of decreased scale-up of ART in the year 2011, this study is being re-evaluated. This study has received funding from the Canadian Institutes of Health Research.

*Discordant Couples (DISCO) Cohort Team* is a four-year cohort study of discordant couples in central-eastern Uganda. It is a partnership between researchers at the BC Centre for Excellence in HIV/AIDS in British Columbia, Canada and The AIDS Support Organisation (TASO) in Uganda [34]. The study follows 550 HIV-uninfected individuals who are co-habiting partners of HIV-infected individuals (i.e. serodiscordant). In 260 of the couples, the HIV-infected partner will be receiving ART, this therefore being the variable being manipulated. The control is 290 couples where the HIV-infected partner has not yet initiated ART (CD4 cell count > 250 cells/mm^3^, WHO stage I or II). This study has received funding from the Canadian Institutes of Health Research.

*An HIV Prevention Program for Mochudi* in Botswana, led by Harvard School of Public Health AIDS Initiative, will determine the feasibility and acceptability of a comprehensive program of interventions, including male circumcision, with a test-and-treat strategy [35]. HIV-positive people with a CD4 cell count above 250 cells/mm^3^ will be offered three-drug ART if they have acute infections and/or their viral load is greater than 50,000 RNA copies/mL, while those with a CD4 cell count below 250 cells/mm^3^ will be referred for ART through the public program. HIV incidence and molecular methods to elucidate transmission pathways will be used to evaluate the impact of the trial which will last for five years (2009-2013).

*The Population Effects of Anti-Retroviral Therapy (PopART/HPTN-071)* study is a cluster randomised trial developed by scientists based at the London School of Hygiene and Tropical Medicine and Imperial College London in the United Kingdom, with partners in South Africa (Western Cape) and Zambia [36]. The purpose of the study is to evaluate the impact of a Universal Testing and Treatment (UTT) intervention on population-level HIV incidence compared to enhanced standard of care in sub-Saharan Africa. The UTT intervention will be delivered as part of a comprehensive combination prevention package and will offer immediate treatment to all those who test positive for HIV irrespective of CD4 cell count. NIH funding has recently been confirmed.

Following a pilot study in 2010, the Africa Centre for Health and Population Studies at the University of KwaZulu-Natal (Africa Centre) [37] is starting the first phase of a cluster-randomised controlled trial,* Treatment-as-prevention (TasP)*, in rural KwaZulu-Natal, South Africa, as part of its overall research objective to test the effectiveness of interventions in reducing HIV incidence in one of the HIV-hyperendemic rural communities in Southern Africa [38]. The trial is funded by the French National Agency for Research on AIDS and Viral Hepatitis (Agence Nationale de Recherche sur le SIDA et les hepatitis virales [ANRS]) and is conducted in collaboration with the University of Bordeaux, the Hôpitaux Universitaires de Genève, and the University of the Mediterranean Aix-Marseille II. The cluster-randomised trial will start in 2011 in four out of the total 32 clusters randomly assigned to either universal ART or standard of care. The primary outcome of the controlled trial is longitudinally-measured HIV incidence; secondary outcomes include the acceptability and feasibility of treatment as prevention, as well as the economic and social consequences.

Doctors Without Borders/Médecins Sans Frontières (MSF) is planning a pilot community-based *Treatment as Prevention (TasP)* project in KwaZulu-Natal, South Africa, in collaboration with the Department of Health, beginning in 2011. The program will aim to reduce HIV and TB incidence, in addition to reducing HIV- and TB-related morbidity and mortality, and to demonstrate the feasibility and acceptability of different approaches to enhanced testing, linkage to care, ART, and retention in care. Pending ethics approval, the project will offer ART for all patients with CD4 counts below 500 cells/mm^3^ and those with CD4 counts above 500 cells/mm^3 ^if their viral load is greater than 100,000 RNA copies/mL. Combination prevention, including medical male circumcision, will be offered and substantial efforts will be made to reduce “leakage” across the test, link, treat, and retain cascade. HIV incidence will be measured by synthetic cohort prevalence surveys at baseline and every 2-3 years.

The Sustainable East Africa Research for Community Health (*SEARCH*) collaboration and University of California, San Francisco are performing pilot studies and community mapping, funded by NIH and the World Bank, in preparation for a community cluster-randomised study of 40 communities to evaluate health (HIV, TB, malaria, maternal mortality), economic and education outcomes of offering treatment to all HIV-infected persons in three East African countries—Uganda, Kenya and Tanzania. Intervention communities will receive annual HIV testing during a community health campaign, and ART will be offered to all HIV-infected children and adults through streamlined care delivery systems. Resource mobilisation in underway for a target start date of November 2012 [39].

*STOP AIDS NOW*! and Clinton Health Access Initiative (CHAI) have been granted Euro 8.8 million for a *Treatment Centered Prevention (TCP)* project in Swaziland from 2011-2014 [40]. Titled *MaxART Maximizing ART for Better Health and Zero New Infections*, the project will ensure that at least 90% of those in need of treatment under current guidelines are on treatment by the end of 2014. The impact of universal access to treatment based on clinical and immunologic criteria on HIV incidence will be evaluated. The study will determine whether a 50% reduction in the number of new HIV infections in Swaziland is possible by 2020.

*Early Antiretroviral Treatment and/or Early Isoniazid Prophylaxis against Tuberculosis in HIV-infected Adults (ANRS 12136, TEMPRANO) study* is being conducted by Université de Bordeaux [41]. This randomised trial will compare the benefits and risks of initiating ART according to the WHO guidelines to the benefits and risks of initiating ART immediately among HIV-infected adults with CD4 counts >350 cells/mm^3^ and <800 cells/mm^3^. In this study, half the patients will also receive six-month isoniazid prophylaxis. This study, to be conducted in Côte d'Ivoire from 2008 – 2013, is sponsored by ANRS.

*Immunology and Outcomes after ART in HIV/TB co-infection* is a prospective cohort study by University of Pennsylvania in Gaborone, Botswana [42]. This five-year (2009-2014) project received funding from NIH. It proposes to evaluate the relationship between very early virologic and immunologic responses to ART and risk of death in the first six months after ART initiation among adults with advanced HIV disease (CD4 count <100 cells/mm^3^) and active TB. This study will help improve the outcomes in global ART scale-up efforts.

*Enhance Prevention in Couples (EPIC)* is a study being conducted by ICAP at Columbia University in collaboration with Ministry of Health in Lesotho aiming to address HIV transmission among discordant couples [43]. The National Institute of Allergy and Infectious Diseases (NIAID) has financed this four-year study to conduct several feasibility/acceptability and modelling studies aiming towards a randomized clinical trial that will evaluate the effect of Enhanced Prevention Package *versus* Standard of Care on risk of HIV acquisition in HIV-negative partners within HIV-discordant couples in Lesotho. 

*Multi-component, targeted HIV Prevention for Sub-Saharan Africa: PreventionRx *is a four-year study by University of Washington that started in 2010 and is receiving financing from NIH [44]. Based on epidemiologic analyses and mathematical modelling of determinants of heterosexual HIV transmission in sub-Saharan Africa and potential impact of targeted preventive interventions, this study will design an evidence-based behavioural and biomedical intervention package to be delivered through a home-based voluntary counselling and testing (VCT) to highest-risk individuals. A community-randomised effectiveness trial of this prevention package (which will include interventions like ART and male circumcision) will be implemented in Eastern and Southern Africa to determine effects of interventions on population-level HIV transmission.

*Interventions to Decrease HIV Infectiousness in Uganda and South Africa* is another University of Washington project being funded by NIH for the period 2010-2013 [45]. It will start in July 2011 and build on the home-based VCT platform (HBCT-plus) in high HIV prevalence areas of Uganda and KwaZulu-Natal. The aim is to increase the knowledge of HIV-positive status with behavioural change, and reduce HIV infectiousness through effective linkages to ART and treatment of co-infections. The performance of the HBCT-plus program will be measured by impact on community viral load and transmission potential.

*HIV/HAART and Pregnancy/Contraception in Rakai, Uganda, *a Johns Hopkins University project from 2009-2012, has received funding from NIH [46]. This study seeks to test how decisions regarding HIV prevention, contraceptive use and pregnancy desires are modified by availability and use of ART and effect of ART on pregnancy outcomes, contraceptive use and HIV risk transmission.

*Impact of HIV, ART and TB Genotype on Survival in Multi-drug Resistant (MDR) TB*, a 5-year study designed by Albert Einstein College of Medicine, has received funding from NIAID starting 2010 [47]. It will take place in rural South Africa and examine the impact of ART on improving survival in MDR TB/HIV co-infection. Furthermore, the study will examine the effect of MDR TB and HIV co-treatment on outcomes for each disease.

*Integrated Prevention Demonstration Campaign (IPD)* was an innovative public health campaign organized by Vestergaard Frandsen, Kenyan Ministry of Health and Cooperative Housing Foundation (CHF) International in the Lurambi division of western Kenya in 2008 [48]. The campaign included community-based voluntary HIV testing, counselling, on-the-spot CD4 cell count and distribution of CarePack, which contained interventions like condoms and educational material. The campaign was successful in reaching 80% of the targeted population in 7 days in an area with a very poor HIV testing rate, and mean CD4 count for HIV-positives was above 560 cells/mm^3^. All people who tested positive were referred for further care. Post-campaign studies are underway to determine the impact of easy access to HIV testing, disease prevention commodities and referral to care services on HIV incidence. Future integrated multi-disease prevention campaigns that include HIV, TB, malaria and other disease prevention research components are targeted for selected countries in sub-Saharan Africa.

*Gender-specific Combination HIV Prevention for Youth in High-Burden Settings (MP3-Youth)* is an NIH-funded study that will determine, mathematically model, and pilot evidence-based intervention prevention packages for male and female youth in western Kenya, including HIV testing and ART referrals [49]. The study is led by New York University in partnership with University of Nairobi and Impact Research and Development Organisation (IRDO), a local non-governmental organization (NGO).

*The Test and Linkage to Care (TLC-IDU) Kenya* study will provide data regarding implementation of the seek, test, treat and retain paradigm with injection drug users (IDUs) in sub-Saharan Africa. The team (led by Kenya’s National AIDS and Sexually Transmitted Infection [STI] Control Programme along with New York University) will conduct a cluster-randomised stepped wedge trial of Kenya’s planned needle exchange program, utilising rapid HIV and point of care CD4 testing and referral to peer ART case managers for people living with HIV with CD4 <350 cells/mm^3^.

*IeDEA Southern Africa *is a large collaborative network of 23 HIV treatment sites in Botswana, Lesotho, Malawi, Mozambique, South Africa, Zambia and Zimbabwe. The current database includes 288,015 adults (251,759 on ART) and 30,470 children (24,277 on ART). The coordination and data centres of IeDEA-South Africa are located in Bern, Switzerland and Cape Town, South Africa. This observational database is used to develop an individual-based, comprehensive mathematical model of the impact of ART on HIV transmission in Southern Africa. In particular, the role of routine viral load monitoring, the impact of the new WHO guidelines and the effect of increasing ART coverage will be explored.

*Swaziland HIV Incidence Measurement Survey (SHIMS)* is designed to be a four-year, population-level HIV incidence study assessing the impact of expanded HIV prevention, care, and treatment activities in Swaziland [50]. The assessment entails two serial, short-term cohorts to estimate and compare HIV incidence rates before and after a national male circumcision campaign, ART scale-up and other prevention activities. Primary outcomes include HIV incidence rates in men and women, HIV incidence rates in circumcised and uncircumcised men, and sexual risk behaviours in high-risk age groups of men and women. The SHIMS study is a joint endeavour of the Swaziland Ministry of Health, the US President’s Emergency Plan for AIDS Relief (PEPFAR) programme in Swaziland, the US Centers for Disease Control and Prevention (CDC), International Center for AIDS Care and Treatment Programs (ICAP) at Columbia University and the University of Washington.

The Zambian Ministry of Health and key partners will implement a National Antiretroviral Programme operational research project focused on the ability of ART sites to implement 2010 adult HIV treatment recommendations to treat HIV-infected adults in discordant relationships and determine patient outcomes. Over the past decade, Zambia’s National HIV Care and Treatment Programme has expanded at a rapid pace. By end of 2010, the country had more than 450 antiretroviral care and treatment facilities, 1200 prevention of mother-to-child transmission of HIV facilities nationwide, and had enrolled more than 500,000 adults and children into long-term care, of whom 344,000 had started ART. The 2010 Zambian Adult & Adolescent HIV Treatment Guidelines include among others, the recommendation to treat all HIV-infected adults in a serodiscordant relationship irrespective of their CD4 count in an attempt to reduce the spread of HIV in the community, particularly among discordant couples. It is estimated that up to 93% of new HIV infections in Zambia occur in couples in a stable cohabiting or marriage relationship [51] resulting in discordancy contributing significantly to the spread of HIV in this population. The national programme intends to monitor the implementation of this recommendation in operational research based in two rural districts. The focus will be on 1) establishing baseline data from these facilities on the number of couples being tested for HIV and what proportion are discordant; 2) using data to improve estimations regarding the proportion of new HIV infections occurring in individuals in a stable cohabiting or marriage relationship; 3) defining the linkage between counselling and testing, and enrollment into ART services and initiation of treatment for the HIV-infected person in a serodiscordant relationship; 4) evaluating the implementation of 2010 HIV treatment recommendations to treat all HIV-infected persons in a discordant relationship and its outcome at facility level; and 5) determining the HIV status of the negative partner through a defined time period for couples accepting ART for their own health as well as preventing the spread to the negative partner. The prospective, observational cohort study of adults accessing testing and counselling as well as ART services in two districts in Zambia will start in late 2011 and end in late 2012. It will use routine programmatic data from registers and electronic medical records in Chongwe and Mumbwa Districts, Zambia.

HIV Prevention Trials Network's *HPTN-070*, *International HIV Testing, Linkage to Care and Treatment *(iTLCT) study [52] is a proposed study to assess in high-prevalence African communities the feasibility of home-based HIV testing; home-based screening and diagnosis of TB in HIV-seropositive persons; strategies for linkage to care; using high plasma viral loads as a criterion for starting ART for those currently not eligible for ART based on prevailing clinical and CD4 guidelines; and estimating HIV prevalence in pregnant women aged 15-19 years attending antenatal clinics in participating communities.

### Region: Asia

*Seek, Test, Treat Strategies for Vietnamese Drug Users: A Randomized Controlled Trial *is a project being undertaken by Johns Hopkins University in the US from the grants from NIH for the period 2010-2015 [53]. This project will intervene with injection drug users by implementing a new approach to HIV testing *via *drug treatment centres, promptly referring HIV-positive people to care, and retaining those individuals on ART in treatment. In addition, behavioural risk reduction interventions will be provided. Using a randomised controlled trial, effects of this seek, test, and treat strategy on ART uptake, ART adherence and treatment outcomes will be evaluated.

At the annual national AIDS meeting in February 2011, China announced efforts to implement a country-wide *HIV Testing as Prevention Strategy and* *ART* *Treatment as Prevention Strategy *as a part of National HIV/AIDS policy [54]. The National Center for AIDS/STD Control and Prevention (NCAIDS) in the Chinese Center for Disease Control and Prevention (China CDC) has collaborated with the British Columbia Centre for Excellence in HIV/AIDS (BCCfE) for this national programme. It is modelled on BCCfE's work in Vancouver, British Columbia. A recently closed study, supported by Chinese Ministry of Health, implemented by NCAIDS and Fudan University, using randomised community trial study design, demonstrated that counselling and condom promotion have hardly changed HIV incidence but antiretroviral treatment of HIV infected individuals has significantly reduced HIV incidence among discordant couples in Dehong, Yunnan, China [55]. Based on the combination of results from this study, recently released *HPTN 052* results and previously published literature, China has decided, in May 2011, to provide ART to some 30,000 discordant couples irrespective of CD4 count as quickly as possible. Given the uncertainty of long-term benefit and risk, an ongoing evaluation of the treating discordant couple programme will be carried out, collaborating with BCCfE, WHO, UNAIDS, and US CDC’s Global AIDS Program (GAP) in China.

Also, NCAIDS and AIDS Care China will operationalise the *Treatment 2.0 *strategy in Wuhan City and Xiangfang City (Hubei) from 2011 onwards, and later on in two other sites in Yunnan and Guangxi with the support of WHO, UNAIDS, the Treatment Access Campaign, CHAI and the Pangaea Global AIDS Foundation. This pilot project focuses on community-based rapid HIV testing and access to quality ART for all medically eligible HIV patients, and will evaluate the impact of expanded access to ART. As a part of the *Treatment 2.0* project, NCAIDS has proposed an impact and cost-effectiveness analysis research plan. One such study on cost-effectiveness of ART on serodiscordant couples will measure the effectiveness and cost-effectiveness between a group receiving ART and a non-ART group and between pre-ART and post-ART in terms of prevention of spousal transmission.

Vietnam is currently designing a *Treatment 2.0 *project that will help the health ministry in planning the country's HIV strategy. The project proposes to adopt a simple service delivery system at two pilot sites in the country and evaluate the cost-effectiveness of this new system. The treatment for prevention component of this project will evaluate the impact of alternate ART initiation criteria on HIV incidence, TB incidence and AIDS-related mortality. The modelling output and cost data will be useful in estimating the resources needed to control HIV/AIDS in a concentrated epidemic situation.

### Region: Europe

*Partners of People on ART: a New Evaluation of the Risks *(*PARTNER)* study is a collaborative effort between Royal Free and Copenhagen HIV Programme (CHIP) and has been funded by the National Institute for Health Research, United Kingdom [56]. This study was approved in 2011 and will be carried out in 55 clinics in 14 countries in Europe. It aims to study HIV-serodiscordant partnerships that report having unprotected sex, to determine the risk of HIV transmission when the HIV-positive partner on ART has plasma viral load <50 copies/mL. Also, factors responsible for non-usage of condoms and for adoption of consistent condom use will be examined. Effectiveness of ART in reducing transmission is expected to be the strongest reason for non-usage of condoms.

### Region: North America

British Columbia Centre for Excellence in HIV/AIDS (BCCfE), Vancouver, Canada has concentrated efforts on mitigating the HIV epidemic in the community. It focuses on hard-to-reach populations including aboriginal peoples, injection drug users, women, and men who have sex with men. Their intervention pilot project will focus on Vancouver and Prince George, British Columbia, and is designed to go beyond these two cities and to other groups. Research efforts are also directed towards HIV risk behaviour and outcomes of ART among injection drug users [57,58,59]. BCCfE’s AIDS Research Program has also focused work on ART regimens in Canada, US and sub-Saharan Africa. Some of their studies on ART include research on optimal timing of ART initiation, effect of ART on adult mortality, and relations between ART adherence and HIV-related mortality [60,61,62,63,64]. BCCfE's current research agenda focuses on *Seek and Treat for Optimal Prevention of HIV/AIDS Initiative* (*STOP HIV/AIDS*) - an initiative to evaluate the 'Treatment as Prevention' strategy in British Columbia. Key projects look at the impact of expanded ART access on HIV/AIDS-related morbidity, mortality, and HIV incidence, and the cost and cost-effectiveness of such strategies [65,66]. British Columbia Ministry of Health has provided major support for the BCCfE’s efforts to decrease HIV incidence in the community. Studies are ongoing and additional studies are slated for publication.

*HAART Optimism, Drug Use and Risky Sexual Behaviour among MSM in British Columbia *is a prospective study by Simon Fraser University for the period 2011-2016 and is financed by NIH [67]. This study examines the effect of a recently initiated population-level intervention (expanded universal and free-of-cost ART as an HIV prevention measure) on HIV risk behaviour among men who have sex with men (MSM) in British Columbia.

*Effect of HAART Expansion on Community Levels of HIV Viral Load and HIV Risk Behaviours Among Men Who Have Sex with Men (MSM) in British Columbia* is a Simon Fraser University project for the duration 2010-2013, being funded by Canadian Institutes of Health Research [68]. It will examine the impact of expansion of access to ART on risk behaviour among the MSM population in Greater Vancouver and on community HIV viral load as a marker of community infectivity.

San Francisco Department of Public Health, California research focuses on HIV health, treatment and prevention outcomes in San Francisco, especially among populations at risk. A community-based study titled *Decreases in Community Viral Load are Accompanied by Reductions in New HIV Infections in San Francisco* [69] was undertaken from 2004-2008 to determine best approaches for HIV prevention and treatment. It hypothesised and concluded that increased HIV testing and ART coverage and effectiveness were associated with decrease in community viral load and hence in new HIV infections. The department publishes data on HIV epidemiology and emerging trends of the HIV epidemic in the city. Studies on community-wide prevention programmes are ongoing to assist the department in forming sound health policies.

*Project HOPE - Hospital Visit as Opportunity for Prevention and Engagement for HIV-infected Drug Users (NIH CTN 0049) *is a multisite, three-arm randomised controlled trial that will compare two distinct approaches to improving outcomes among hospitalised substance-using HIV patients [70]. The study will include 800 patients at ten sites in urban centres across the US that are heavily affected by HIV, and will focus on viral suppression as the primary endpoint. In addition, the study will also determine linkage and retention in HIV primary care, linkage and retention in drug abuse treatment, and reduction in numbers of hospitalisations. Participants will be randomised to one of three groups: 1) an active patient navigator component: a strengths-based case management approach that includes motivation, physical escort to treatment, and face-to-face booster sessions; 2) a passive incentives/contingency management component to further motivate and reinforce completion of target behaviours; or 3) treatment as usual. Enrollment is pending and participants will be followed over a 12-month period.

*HPTN 065: A Study to Evaluate the Feasibility of an Enhanced Test, Link to Care, Plus Treat Approach for HIV Prevention in the United States (TLC- Plus Study) *[71] is being conducted by the HIV Prevention Trials Network (HPTN) in several communities in the US. Two communities serve as intervention communities (Washington, District of Columbia [DC] and the Bronx, New York) and four non-intervention communities (Chicago, Illinois; Houston, Texas; Miami, Florida; and Philadelphia, Pennsylvania). The purpose of the study is to evaluate the feasibility of an enhanced community-level “test, link to care, plus treat” strategy in the US [71]. The study includes the following components. Each component of the study involves an independent design but is interrelated to the other components: 1) Expanded HIV Testing component: involves social mobilisation, with targeted messaging to promote testing, and implementation of the universal offer of HIV testing in emergency departments and hospital inpatient admissions; 2) Linkage-to-Care and Viral Suppression components: involves site randomisation to test the effectiveness of a financial incentive (FI) intervention compared with the standard of care (SOC); 3) Prevention for Positives component: uses individual randomisation to compare the SOC plus a computer-delivered intervention with the SOC and; 4) Patient and Provider Survey component: administered at specific time points during the study to assess knowledge, attitudes and practices regarding early initiation of ART and the FI interventions. This study is funded by NIH and the US CDC and is being implemented in collaboration with Columbia University and local health departments. The study period is September 2010 to February 2014.

A *Randomized Controlled Trial of HIV Testing and Linkage to Care*, undertaken by Friends Research Institute and The Miriam Hospital-Lifespan is being conducted in community-based corrections facilities at two sites – Providence, Rhode Island and Baltimore, Maryland in the US [72]. First, a randomised controlled trial of HIV testing will study the efficacy of on-site rapid testing at a probation/parole office versus off-site referral at a community health centre or HIV testing clinic. The second study is a randomised trial wherein all individuals identified at community corrections with HIV will be offered enrollment in a one-year intervention study to examine the ability to improve linkage into HIV care. Participants will be randomised to receive one of two conditions: 1) Project Bridge (a case management-based strategy) for one year, or 2) Treatment as Usual (referral to standard level of care). Moreover, those randomised to treatment as usual will be given an opportunity to cross over to Project Bridge if they have failed to engage in treatment during the first three months. This project has been financed by NIH for the period 2010-2015.

*Effectiveness of Peer Navigation to Link Released HIV+ Jail Inmates to HIV Care* study is being conducted by University of California, Los Angeles among HIV-positive male ex-inmates who are being released from Los Angeles County Jail system [73]. The aim of this five-year NIH-funded study is to examine the individual and structural barriers to HIV care after release from jail and to utilize a randomised design to evaluate the impact of a peer-based health system navigation intervention on linkage and retention in care, ART adherence and viral load suppression.

Miriam Hospital (Brown University, Providence, Rhode Island) is working on *Improving Linkage to HIV Care Following Release from Incarceration* by designing and implementing a monitoring strategy to evaluate follow-up HIV care in community post-release [74]. This study will test the clinical and epidemiological utility of this strategy and identify individual, community, institutional and political factors influencing linkage to HIV medical care during the pre- and post-release periods. The project has also received NIH grants for a five-year period starting 2010.

University of North Carolina, Chapel Hill has received a five-year NIH grant for their study *Randomized Control Trial of an Augmented Test, Treat, Link, and Retain Model for North Carolina and Texas Prisoners *[75]. This ongoing trial compares a comprehensive multi-component intervention that spans incarceration and release, and includes motivational interviewing and a brief link coordination based on the University of Alabama’s Project Clinic Oriented New patient Navigation to Encourage Connection to Treatment (CONNECT) model with standard discharge planning for HIV-infected prisoners. The study will be conducted in North Carolina and Texas (which combined incarcerate 15% of all those in prison in the US). Participants will be about-to-be-released inmates with suppressed plasma HIV RNA levels. The primary aim is to determine the effect of the intervention on maintaining suppression of viraemia post-release *via *adherence to ART and engagement in ongoing HIV care. Secondary outcomes include risk behaviour following release.

*Seek, Test, Treat: An Integrated Jail-Prison-Community Model for Illinois *is being developed by University of Illinois in the US [76]. The project has received funds from NIH for five years starting in 2010. It constructs and evaluates a seek, test, treat (STT) model that begins at entrance to jail, continues through prison and extends into the community after release. STT has five components: 1) opt-out HIV testing in jail and prison, 2) transition case management for HIV-positive persons leaving jail and prison, 3) university-based telemedicine for all state prisoners living with HIV and HIV specialty care from jail-based staff, 4) incentives to visit community-based organizations following release from jail, and 5) social network HIV testing and partner notification. The study will also evaluate the overall impact of this model on community-level HIV viral load.

*Seek, Test, and Treat Strategies* study is led by Center for AIDS Intervention Research, Medical College of Wisconsin, US [77]. It was one of the twelve studies to receive grants from NIH for the period 2010-2015. The study will evaluate the cost and cost-effectiveness of a comprehensive and systematically coordinated network approach of HIV testing referral. This approach will test people in a state correctional facility being released to a major metropolitan area, (re)link HIV-positive people into low- or no-cost treatment and case management services, and seek to evaluate an innovative network method of HIV testing referral to utilise high-risk HIV-negatives in the correctional system to encourage other high-risk individuals in their social network to get tested.

*CARE Corrections: Technology for Jail HIV/HCV Testing, Linkage, and Care* (*TLC*) Study is a research project being conducted by Miriam Hospital/Brown University, New York University, and George Washington University and is being supported by NIH for five years [78]. The *TLC* study modifies two information and communication technology-based tools, CARE and CARE+, for use among jail detainees in Rhode Island and Washington, DC. Using randomised controlled trials, this project will evaluate the effectiveness of CARE Corrections-delivered counselling to facilitate linkage to community HIV care, maintain HIV viral suppression, and decrease HIV transmission behaviours. The project will evaluate the cost-effectiveness of the CARE Corrections tools compared to standard of care services.

*Finding, Testing and Treating High-risk Probationers and Parolees with HIV* is a study by Research Triangle Institute to be conducted in Oakland, California [79]. It received grants from NIH in 2010 for five years. This project seeks to design and implement a community-based strategy of HIV testing and counselling for drug users on probation or parole, and assess the efficacy of Project Bridge compared with a usual care for HIV-positive patients using a randomised controlled trial. Outcomes of interest will be the proportion of eligible individuals who are identified and recruited, accept HIV testing, have not been HIV tested in the previous six months, and report recent HIV risk behaviour.

*START Together: HIV Testing and Treatment in and after Jail *is a five-year NIH-funded project focused on HIV prevention, testing and treatment for individuals in jails [80]. It is undertaken by National Development and Research Institutes at Rikers Island facilities in New York City. *START Together* has 3 components: 1) Project START (an HIV reentry program), 2) Computer Assessment and Risk-Reduction Education and 3) Peer Health Navigators. The study seeks to test whether *START Together* increases the proportion of inmates receiving HIV testing and the proportion of individuals with undetectable HIV viral load post-release.

The use of medication-assisted therapy (MAT) to enhance ART for soon-to-be-released HIV-infected criminal justice system (CJS) populations in Connecticut and Massachusetts is the focus of two NIH-funded grants by Yale School of Medicine researchers. The main hypothesis is that the prevention of relapse to drug and alcohol use *via *MAT will improve linkage to care for HIV-infected released CJS populations and will lead to greater HIV viral load suppression, thereby decreasing individual morbidity as well as decreasing transmission to the uninfected public. Both studies are five-year randomised, placebo-controlled trials of extended-release naltrexone, an opioid antagonist that is approved by the US Food and Drug Administration (FDA) for the treatment of opioid and alcohol dependency, among released HIV-infected CJS populations. The first study funded by the NIH,* Alcohol Pharmacotherapies Among Released HIV+ Prisoners, *is among prisoners in Connecticut transitioning to the community with alcohol use disorders and will assess HIV treatment, alcohol treatment and HIV risk behaviour outcomes. The second study is funded by NIH as one of the twelve *Seek, Test, and Treat: Addressing HIV in the Criminal Justice System* sites and is entitled *Naltrexone for Opioid Dependent Released HIV+ Criminal Justice Populations.* This study will take place in Massachusetts and Connecticut; HIV treatment, opioid treatment and HIV risk behaviour outcomes are examined among prisoners and jail detainees who are being released to the community. Outcomes from these studies will establish the efficacy, safety and tolerability of MAT as an effective, evidence-based treatment for opioid and alcohol dependence for released HIV+ CJS populations that can improve viral load suppression *via *preventing relapse to drug and alcohol use, thereby improving adherence to ART and HIV care.

*The Peer-driven Intervention to Seek, Test & Treat Heterosexuals at High Risk for HIV (HHR)* study will use National HIV Behavioral Surveillance System methodology to target HHR and overcome structural barriers to HIV testing and treatment. The primary goal of the proposed study is to evaluate the efficacy of a multi-level enhanced peer-driven intervention to seek, test, treat and retain HHR in New York City.

*HIV, Buprenorphine, and the Criminal Justice System* is a Yale University study that received grants from NIH for 2010-2015. It focuses on incarcerated, opiate-addicted individuals transitioning back to the community from prison or jail in Washington, DC. Opioid relapse in this group is associated with decreased ART adherence, ART discontinuation, loss in viral suppression within three months post-release and increased HIV risk behaviour. The efficacy of buprenorphine to improve adherence and retention in both drug abuse treatment and HIV care, and to reduce HIV transmission will be examined in a placebo-controlled randomised clinical trial.

### Region: Global

International Network for Strategic Initiatives in Global HIV Trials (INSIGHT)'s *Strategic Timing of Antiretroviral Treatment* (START) is an international randomised trial in 37 countries to determine if the chance of developing a serious illness or of getting AIDS is less if patients start taking HIV medicines at a time when their CD4 cell count is above 500 cells/mm^3^ rather than waiting for it to drop to 350 cells/mm^3^ [81]. The study plans to enroll 4000 participants by the end of 2012 and follow them through 2015. The study, which is funded primarily by NIAID, will also examine how early ART will affect chances of developing resistance to HIV medicines, quality of life, health care utilisation and the cost of medical care.

*Test & Treat to End AIDS (TTEA)* is an international group of scientists, doctors and evaluation experts that is advocating for the US congress and executive branch of the US government to adequately fund, implement and evaluate several large-scale test and treat “implementation research” projects in three or more PEPFAR countries disproportionally affected by HIV/AIDS [82]. The purpose of these “implementation research” projects would be to determine the strategy’s effectiveness (in light of the *HPTN 052* results) in significantly reducing and/or eliminating HIV transmission at the population level, significantly reducing long-term pandemic costs, saving lives, and providing donors an exit strategy from having to continually fund HIV. The start date of the advocacy project is between May 1, 2011 and April 30, 2012.

The *Reducing Early Mortality and Early Morbidity by Empiric Tuberculosis Treatment Regimens* (*REMEMBER*) study (*ACTG 5274*) is a randomised, open-label, phase IV strategy trial that will be conducted in Africa, Asia, South America, and the Caribbean in the 18 international AIDS Clinical Trials Group (ACTG) sites. This 96-week study will start in 2011 and be conducted in resource-limited settings with a high regional tuberculosis (TB) incidence (i.e. the TB incidence within the site’s expected catchment area) of more than 100 cases/100,000 population per year, a national ART programme, and documented high mortality rates among HIV-infected individuals (i.e. at least 5% overall at six months post-ART initiation). It includes participants from resource-limited settings who present with advanced HIV disease and no probable or confirmed TB, as defined in the current ACTG diagnosis appendix. Participants who are initiating ART will be randomised to one of two strategy arms: immediate, empiric TB treatment (public health approach) or local standard of care TB treatment (individualised approach). The primary endpoint is survival status in the two arms 24 weeks after randomisation. AIDS progression (any new WHO Stage 3 or 4 condition), virologic and CD4 cell response, HIV and TB drug resistance, and safety and tolerability of and adherence to HIV and TB drugs will be evaluated, as will the cost-effectiveness of the two strategies. Patients with probable or confirmed TB at screening will be excluded. The study is funded by the NIH and primary results are expected in 2014.

## DISCUSSION

There is considerable scientific evidence supporting the use of ART in prevention of HIV and TB and a growing number of ongoing and planned research activities in this area [9,10,83]. Using a limited search methodology we found 50 projects representing 5 regions and 52 countries examining ART in prevention with a global distribution. There are 24 randomised controlled clinical trials with at least 12 large randomised individual or community cluster trials in resource-constrained settings that are in the planning and early implementation stages. Although by definition the studies focus on using ART to prevent HIV and TB, there is considerable heterogeneity between studies in terms of methodology, interventions (e.g. ART eligibility) and geographical location. Additionally, while the identified studies will undoubtedly advance our understanding of the efficacy and effectiveness of ART for prevention, some key questions may remain unanswered or only partially answered. For example, there are only a few randomised trials in resource-constrained settings looking at the issue of when to start ART and the population impact of expanded access to earlier ART. The large number and wide variety of research projects emphasise the importance of this research issue and clearly demonstrate the potential for synergies, partnerships and coordination across funding agencies.

There is considerable urgency to find effective solutions to prevent the 7000 new HIV infections that are estimated to occur each day. While many of the research studies will provide information over the near term, many of the key trials are in the early stages and will likely take at a minimum of two to four years for preliminary results to become available. The majority of community-based studies in resource-constrained settings are observational or prospective cohort design. Although data from observational studies is of considerable value, the WHO and other guidelines development processes favour randomised controlled trials (RCT) [84-86]. The 20-year time frame from early observational to RCT data before issuing recommendations for male circumcision provides a cautionary tale regarding potential delays in the research to policy process—in this case, most likely due to policy makers requiring evidence from RCTs before changing guidelines and developing policy. Many questions specific to ART in prevention of HIV and TB are amenable to a less expensive and more rapid operational research approach that uses data from current ART expansion efforts to answer relatively simple but important questions. For example, some research groups have opted to study operational questions and impact during district- or province-wide expansion of access to ART at higher than recommended levels. The rapid expansion of ART in resource-limited settings has been accompanied by an unprecedented collection of data on a wide variety of indicators. Although our search methodology did not specifically identify them, we anticipate the publication of retrospective analyses focusing on existing program and research data. The difficulty and delays inherent in implementing RCTs will likely mean that near- and medium-term policy decisions will necessarily need to rely on observational data, often from studies implementing combination prevention approaches. Reminiscent of infection control for TB [87,88], we may be faced with recommending a combination of evidence-based interventions without being able to attribute the decrease in transmission to a single intervention. Given the urgency of the situation, a blend of research methodologies that answer key questions resulting in near-, medium- and long-term evidence will likely be the most useful to policy makers, public health authorities and affected communities.

Our study had a number of limitations. While we used a standardised methodology and thorough review of listed studies, our search likely missed many planned or ongoing studies. This is particularly true for studies in the planning phase, as these sorts of efforts are often not widely publicised. Our search was done in English which may have excluded key projects in other languages. This is evident in the lack of studies from the South American region. Additionally, it was difficult to determine from brief descriptions of some studies whether they qualified as ART in prevention of HIV and TB studies and the funding amounts. We also did not include the growing list of published ART in prevention of HIV and TB studies. For example, the recently halted *HPTN 052* study was not included, and we also did not include any ongoing or planned modelling research as we opted to focus on empiric studies. Bias may have been introduced through our use of experts to augment our list—as they were predominantly from wealthier and English-speaking settings. Understanding the operational aspects of providing HIV testing and counselling, HIV care including ART, and retention in care is critical, and our limited approach undoubtedly excluded important research in these critical areas. Finally, although basic and behavioural sciences have made considerable contributions to our understanding of the role of ART in prevention, with a few exceptions we did not include most of these sorts of studies in our list. Despite these limitations, our list is the first of its kind and provides considerable insights into the categories, duration, and location of ongoing and planned ART in prevention HIV and TB studies. We anticipate that it will engender further discussion and are hopeful that researchers will contact us for inclusion into future updates of this review. WHO is currently collecting research protocols and concept notes; email requests can be sent to the corresponding author to share a protocol and/or for more information.

Human rights are inviolable and expansion of HIV services should be done within a human rights framework [89]. Another area that has considerable importance but that may have been missed by our approach is research on the ethical and human rights aspects of expanding HIV services. Preliminary work exploring the programmatic costs of integrated human rights and community support interventions in South Africa has previously been presented elsewhere [90], and is included in this issue of *Current HIV Research* (Jones *et al.*). Additional research is needed to explore the potential risks and benefits of human rights and community support interventions. This will be particularly important for the benefits in terms of reaching individual and public health targets, as the potential risks are better described. While voluntary HIV testing and counselling programmes reaching millions of people have been implemented worldwide, monitoring of the quality of testing programmes including the respect afforded to human rights and consequences following testing have rarely been emphasised, and little programmatic or research data have been published. Access to high quality HIV counselling and testing is a fundamental aspect of the right to health care; however, efforts to expand HIV testing to a wide range of different populations have sometimes had serious problems, including violating individual consent and failure to provide confidentiality protections [91-96]. The possibility and consequences of these abuses and efforts to criminalise HIV affects both the research ethics of the above studies, whether community-based or in closed settings [95-97], and the success of ensuring a continuum from voluntary testing to long-term adherence to treatment (see Barr *et al.*, this issue of *Current HIV Research*). Although we did not identify any ongoing or planned research on this important issue, we expect that research on the risks and benefits of expanding ART with and without specific human rights and community interventions will be forthcoming.

While it is increasingly clear that ART is both life-saving and a powerful prevention intervention, there are a number of open policy questions that remain unanswered. Key questions including when to start ART; the potential impact of ART on HIV and TB prevention, feasibility, acceptability, and cost; and how to achieve improved coverage are all being actively researched. Although a few important randomised controlled trials in resource-constrained settings are in the planning or implementation stages, it is highly likely that key policy decisions will need to be made using the available near- and medium-term observational data derived from programme data or specific research studies. Despite the multiplicity of research around the broad theme of ART in prevention of HIV and TB, the numerous and potentially divergent study outcomes and the staggered timelines may challenge the ability of normative agencies and countries to issue guidance in a timely manner. WHO and UNAIDS will continue to work with key stakeholders to map outstanding research issues, encourage collaboration among researchers and the community, and foster the coordination and rapid evaluation and incorporation of new evidence into policy.

## Figures and Tables

**Table 1.** List of Ongoing and Planned Research Projects on Antiretroviral Therapy (ART) in Prevention of HIV and Tuberculosis (TB)

Project	Study Type	Focus	Principal Interventions	Outcomes	Region	Institution	Time Period
					AFRICA		
Scaling Up Treatment to Reduce Population Level Incidence of HIV/AIDS	Step-wedge community level randomised trial	Impact of ART scale-up on HIV incidence	Increased access to ART	HIV and TB incidence, HIV- and TB-related mortality	Uganda	University of British Columbia, Joint Clinical Research Centre	2011-2015
Discordant Couples (DISCO) Cohort Team	Cohort study	Impact of ART on partner acquisition of HIV	ART for HIV positive partner	Risk of HIV acquisition in HIV negative partner	Central-eastern Uganda	University of British Columbia, The AIDS Support Organization	2009-2012
An HIV Prevention Program for Mochudi, Botswana	Population-based observational study	Implementation of effective behavioural and biomedical preventive interventions	Opt-out HIV testing, ART for high viral load, behaviour modification education and male circumcision	HIV incidence, HIV transmission	Mochudi, Botswana	Harvard University School of Public Health, USA	2009-2013
(Population Effects of ART) Trial (HPTN071)	Randomized controlled trial	Preventive effects of universal testing and treatment (UTT) intervention	ART irrespective of CD4 cell count	Population-level HIV incidence	South Africa (Western Cape) and Zambia	London School of Hygiene and Tropical Medicine, Imperial College London, UK	Funded (Planning Phase)
TasP (Treatment as Prevention)	Cluster-randomised controlled trial	Effectiveness of treatment-as-prevention in reducing HIV incidence in a general population	Widespread HIV testing and ART irrespective of CD4 count	Population-level HIV incidence	Rural KwaZulu-Natal, South Africa	Africa Centre for Health and Population Studies, University of KwaZulu-Natal; Université Bordeaux, Hopitaux Universitaires de Geneve; ANRS sponsor	2011 onwards
TasP (Treatment as Prevention)	Prospective cohort study	Feasibility and acceptability test, link, treat and retain strategy	ART at CD4 count < 500 cells/mm3, male circumcision, and test, link, treat and retain strategy	HIV and TB incidence, HIV- and TB-related morbidity and mortality	KwaZulu-Natal, South Africa	Medecins Sans Frontieres (MSF)	2011 onwards
Sustainable East Africa Research for Community Health (SEARCH)	Community cluster- randomised trial	Health, economic and education outcomes of community health campaign providing HIV testing and treatment services	Annual HIV testing, ART for all CD4 cell count, streamlined care	HIV incidence; TB, malaria, maternal, HIV and all-cause mortality; education and economic outcomes	Uganda, Kenya, Tanzania	University of California, San Francisco, USA, International Development Research Centre (IDRC), Kenya Medical Research Institute (KEMRI)	2010 onwards
MaxART Maximizing ART for Better Health and Zero New Infections	Population-based observational study (entire country)	Universal ART following concept of *Treatment* 2.0 within 3 years	ART access for 90% of people with HIV eligible for treatment	HIV incidence, TB incidence	Swaziland	STOP AIDS NOW!, Clinton Health Access Initiative (CHAI) Swaziland, Ministry of Health, Nercha, Swannepha, SafAIDS, GNP+, University of Amsterdam, SACEMA and Dutch Postcode Lottery	2011-2014
Early Antiretroviral Treatment and/or Early Isoniazid Prophylaxis against TB in HIV-infected Adults (TEMPRANO Trial)	Randomised controlled trial	Benefits and risks of early ART in HIV-infected people	Early ART before CD4 count reaches 350 cells/mm3 and 6 months of isoniazid	Death (all-cause), AIDS-defining disease, non-AIDS-defining malignancy, or non-AIDS-defining invasive bacterial disease	Abidjan, Cote d'Ivoire	Université Bordeaux, France, Treichville Université hôpital, Abidjan, Côte d’Ivoire, ANRS Sponsor	2008-2012
Immunology and Outcomes after HAART in HIV/TB Co-infection	Cohort study	Relationship between early responses to ART and risk of death among individuals with advanced HIV disease and active TB	ART in advanced HIV infection	Risk of death in first 6 months after ART initiation	Gaborone, Botswana	University of Pennsylvania, USA	2009-2014
Enhance Prevention in Couples (EPIC)	Randomised controlled trial	Effects of enhanced prevention package for serodiscordant couples	Early ART, counselling, male circumcision	Risk of HIV acquisition in HIV- negative partner	Lesotho	ICAP, Columbia University, USA	2009-2013
Multi-component, Targeted HIV Prevention for Sub-Saharan Africa: PreventionRx	Randomised controlled trial	Effects of evidence-based behavioural and biomedical preventive interventions	ART, male circumcision, behavioural interventions	Population-level HIV transmission	Eastern and Southern Africa	University of Washington, USA	2010-2011
Interventions to Decrease HIV Infectiousness in Uganda and South Africa	Community randomised trial	Effectiveness of home-based voluntary counselling and testing (VCT) platform	Enhanced HIV testing, behavioural interventions, effective linkages to ART and treatment of co-infections	Community viral load and transmission potential	Uganda, South Africa	University of Washington, USA	2010-2013
HIV/HAART and Pregnancy/Contraception in Rakai, Uganda	Community-based observational study	Impact of ART on decisions regarding HIV prevention, contraceptive use and pregnancy	ART	Contraceptive use, fertility outcomes, HIV risk behaviour	Rakai, Uganda	John Hopkins University, Baltimore, USA	2009-2012
Impact of HIV, ART and TB Genotype on Survival in Multi-drug resistant (MDR) TB	Cohort study	Impact of ART on survival in MDR TB/HIV co-infection	ART	Mortality in MDR TB/HIV co-infected people	South Africa	Albert Einstein College of Medicine, USA	2010-2015
Integrated Prevention Demonstration Campaign (IPD)	Campaign population-based observational study (province- wide)	Campaign-based approach to testing and referral to care	Early access to HIV testing, counselling, distribution of disease prevention commodities and referral to care	HIV prevalence, feasibility of rapid mass multi-disease prevention campaign	Lurambi, Kenya	Vestergaard Frandsen, Switzerland	2008-ongoing
Gender-specific Combination HIV Prevention for Youth in High-Burden Settings (MP3-Youth)	Cohort study	Effectiveness of gender-specific youth HIV prevention package	Gender-specific interventions - MP3-Youth	Prevention method uptake, adherence, risk compensation behaviour	Western Kenya	New York University, USA and University of Nairobi, Impact Research and Development Organisation, Kenya	2011-2015
Test and Linkage to Care (TLC-IDU) Kenya	Step-wedge cluster-randomised trial	Implementation research on seek, test, treat and retain paradigm with IDUs	Needle exchange programme, rapid HIV testing and point of care CD4 testing, peer treatment	Efficacy of seek, test, treat and retain strategy	Kenya	New York University, USA, Kenyan National AIDS and STI Control Programme	Planning Phase
Scale-up of Antiretroviral Therapy and Transmission of HIV in Southern Africa	Cohort study	Impact of large- scale testing and early ART strategy, and role of routine viral load monitoring	Increased ART coverage and early ART (according to WHO guidelines)	Community-level HIV viral load; HIV incidence	Botswana, Malawi, Mozambique, South Africa, Zambia, Zimbabwe	IeDEA Southern Africa	2011 onwards
Swaziland HIV Incidence Measurement Survey (SHIMS)	Population-based study	Impact of HIV prevention, care and treatment activities	Male circumcision, ART scale-up	HIV incidence, sexual risk behaviours	Swaziland	Swaziland Ministry of Health, PEPFAR, CDC, ICAP, Columbia University, University of Washington	2011-2014
Ability of ART Sites to Implement 2010 Adult HIV Treatment Recommendation to Treat HIV+ Adults in Discordant Relationships and Determine Patient Outcomes	Cohort study	Early ART for HIV-infected adults in discordant relationship as a preventive strategy	ART irrespective of CD4 cell count for HIV-infected partner in discordant couple	New HIV infections	Chongwe and Mumbwa, Zambia	National Antiretroviral Programme, Zambia	2011-2012
HPTN 070: International HIV Testing and Linkage to Care and Treatment (iTLCT) Study	Feasibility study for a community randomised trial	Enhanced testing, treatment and linkage to care strategy versus standard of care in resource-limited settings	Home-based HIV testing, home-based TB screening, linkage to care, ART for people with high viral load	Feasibility of enhanced HIV and TB testing, treatment and linkage to care strategy, HIV transmission	Multi-site study in Africa	NIH, NIAID, HPTN	Planning Phase
					**ASIA**		
Seek, Test, Treat Strategies for Vietnamese Drug Users: A Randomized Controlled Trial	Randomised controlled trial	Effectiveness of seek, test, treat model for injection drug users	HIV testing in drug treatment centers, referral to care and retention of people on ART in treatment	ART uptake, ART adherence, treatment outcomes	Hanoi, Vietnam	John Hopkins University, Baltimore, USA	2010-2015
HIV Testing as Prevention Strategy and ART Treatment as Prevention Strategy	Population-based observational study (selected cities)	Prevention effects of ART in serodiscordant couples	ART for serodiscordant couples irrespective of CD4 count	HIV incidence among serodiscordant couples	China	National Center for AIDS/STD Control and Prevention (NCAIDS), China Center for Disease Control and Prevention (CDC), British Columbia Centre for Excellence in HIV/AIDS, Canada	2011 onwards
Treatment 2.0 Project in China	Population-based observational study (selected cities)	Cost and cost-effectiveness of community-based HIV testing and treatment strategies	Rapid HIV testing, expanded access to quality ART	Cost and cost-effectiveness of Treatment 2.0 project, cost and cost-effectiveness of ART for serodiscordant couples	Wuhan City and Xiangfang City, Hubei, China	NCAIDS and AIDS Care, China	2011 onwards
Treatment 2.0 Project in Vietnam	Population-based observational study (selected provinces)	Optimal time for ART initiation and effects of alternate service delivery systems	Early ART, simple service delivery system	HIV incidence, TB incidence, AIDS-related mortality, cost and cost-effectiveness of simple service delivery system	Vietnam	Ministry of Health, WHO, other stakeholders	Planning Phase
					**EUROPE**		
Partners of People on ART: a New Evaluation of the Risks (PARTNER Study)	Observational study	Risk of HIV transmission in serodiscordant couples on ART who do not use condoms	ART, condom use	HIV transmission risk	14 European countries	Copenhagen HIV Programme (CHIP), Denmark Royal Free University College Medical School, UK	2011 onwards
					**NORTH AMERICA**		
Association of Highly Active Antiretroviral Therapy Coverage, Population Viral Load, and Yearly New HIV Diagnoses in British Columbia, Canada: a Population-based Study	Population-based observational study	Relation between ART coverage, HIV-1 viral load and HIV transmission	ART coverage, viral load, CD4 count	New HIV diagnoses per year	British Columbia, Canada	British Columbia Centre for Excellence in HIV/AIDS, Canada	2009-ongoing
Effect of Early Versus Deferred ART for HIV on Survival	Observational Study	Survival benefits of early ART initiation for asymptomatic patients	ART at CD4 count >350 cells/mm^3^ and CD4 count >500 cells/mm^3^	Relative risk of death	United States and Canada	British Columbia Centre for Excellence in HIV/AIDS, Canada	2009-ongoing
HAART Optimism, Drug Use and Risky Sexual Behaviour among men who have sex with men (MSM) in British Columbia	Population-based observational study	Effects of expanded universal and free-of-cost ART as a preventive measure for high-risk population	Universal and free ART access	HIV risk behaviour among men who have sex with men	British Columbia, Canada	Simon Fraser University, Canada	2011-2016
Effect of HAART Expansion on Community Levels of HIV Viral Load and HIV Risk Behaviours among MSM in British Columbia	Population-based observational study	Effects of expanded access to ART on HIV risk behaviour and viral load	Universal and free ART access	HIV risk behaviour among MSM, HIV viral load	Vancouver, British Columbia, Canada	Simon Fraser University, Canada	2010-2013
Decreases in Community Viral Load are Accompanied by Reductions in New HIV Infections in San Francisco	Population-based observational study	Relation between community viral load and new HIV infections	Increased HIV testing, ART coverage and effectiveness	Annual number of newly diagnosed HIV cases	San Francisco, California, USA	San Francisco Department of Public Health, USA	2004-ongoing
Project HOPE (NIH CTN 0049) -- Hospital Visit as Opportunity for Prevention and Engagement for HIV-infected Drug Users	Randomised controlled trial	Compare two approaches to improving outcomes among hospitalised substance-using HIV patients	1) an active patient navigator component, 2) a passive incentives/contingency management component, 3) treatment as usual	Viral suppression, reducing all-cause mortality, increasing linkage to and retention in HIV primary care, increasing linkage to and retention in drug abuse treatment, and reducing hospitalisations	Multi-site, USA	University of Miami Miller School of Medicine	Planning enrollment (12 month study, 800 patients)
TLC+ (HPTN 065): A Study to Evaluate the Feasibility of an Enhanced Test, Link to Care, Plus Treat Approach for HIV Prevention in the United States	Community-based study	Feasibility of test, link-to-care and treat strategy	Expanded HIV testing, linkage to HIV care and viral suppression, a computer-delivered prevention for positives intervention, and surveys of patients and clinicians	Viral suppression, expanded testing, linkage to care	Washington DC, the Bronx, New York, USA	Columbia University and CDC, USA	2010-2014
A Randomized Controlled Trial and Cohort Study of HIV Testing and Linkage to Care	Randomised controlled trial and cohort study	Efficacy of test and link-to-care strategy at community correction	On-site rapid HIV testing and 1-year Project Bridge	HIV testing, retention in care, ART initiation, HIV plasma viral load	Providence, Rhode Island and Baltimore, Maryland in USA	Friends Research Institute and The Miriam Hospital-Lifespan, USA	2010-2015
Effectiveness of Peer Navigation to Link Released HIV+ Jail Inmates to HIV Care	Randomised controlled trial	Peer-based navigation versus usual care for HIV+ released inmates	Individually delivered peer-based learning approach to address barriers to and facilitators of HIV care retention	Barriers to HIV care, linkage and retention in care, ART adherence, viral load suppression	Los Angeles, USA	University of California, Los Angeles, USA	2010-2015
Improving Linkage to HIV Care Following Release from Incarceration	Observational study	Design, implement and test monitoring strategy for HIV+ ex-inmates to improve linkage to care	Monitoring strategy for follow-up HIV medical care	Individual, community, institutional and political factors influencing linkage to care and ART outcomes	USA	Miriam Hospital, Brown University, Providence Rhode Island, USA	2010-2015
Randomized Controlled Trial of an Augmented Test, Treat, Link, & Retain Model for North Carolina and Texas Prisoners	Randomised controlled trial	Multi-component intervention programme for prisoners pre- and post-release	Mandatory or opt-out HIV testing, universal ART access, personalised linkage to care and support services	Plasma HIV RNA, HIV transmission risk behaviour, incident sexually transmitted infections (STIs), adherence to ART	North Carolina and Texas, USA	University of North Carolina, USA	2010-2015
Seek, Test, Treat: An Integrated Jail-Prison-Community Model for Illinois	Community-based observational study	Effectiveness of seek, test, treat model (STT) that begins in jail and extends into community post- release	Opt-out HIV testing in jails, transition case management, university-based telemedicine, incentives for retention in care and social networking	Community-level HIV viral load	Illinois, USA	University of Illinois, Chicago, USA	2010-2015
Seek, Test, and Treat Strategies	Community-based	Seek, test, treat model (STT) for correctional populations	HIV testing for high risk population, re-link to low- or no-cost treatment services, HIV testing referral for high-risk negative individuals and their networks	Cost and cost-effectiveness of entire STT model and its individual components	USA	Medical College of Wisconsin, USA	2010-2015
CARE Corrections: Technology for Jail HIV/HCV Testing, Linkage, and Care (TLC)	Randomised controlled trial	Use of information and communication tools (ICT) with discharge planning for jail detainees	CARE and CARE+	HIV viral suppression, HIV transmission behaviours and cost-effectiveness of CARE and CARE+	Rhode Island and Washington DC, USA	Miriam Hospital/Brown University, New York University, George Washington University, USA	2010-2015
Finding, Testing and Treating High-risk Probationers and Parolees with HIV	Randomised controlled trial	Community-based seek, test, treat model for drug users on probation or parole	Expanded HIV testing and counselling, Project Bridge	Proportion of eligible individuals recruited, tested and HIV risk behaviour	Oakland, California, USA	Research Triangle Institute, North Carolina, USA	2010-2015
START Together: HIV Testing and Treatment in and after Jail	Randomised controlled trial	Efficacy of comprehensive intervention package START Together in criminal justice system	HIV reentry program for incarcerated populations, computer assessment and risk-reduction education, peer health navigators	Proportion of inmates receiving HIV testing and proportion of individuals with undetectable HIV viral load post-release	New York City, USA	National Development and Research Institutes, New York City, USA	2010-2015
Alcohol Pharmacotherapies among Released HIV+ Prisoners	Randomised controlled trial	Effect of depot-naltrexone for alcohol- dependent HIV- positive prisoners transitioning to the community	Medication assisted therapy - Depot-naltrexone	HIV-1 RNA level, CD4 count, retention in care, alcohol treatment outcome, HIV risk behaviour, adverse side effects	Connecticut, USA	Yale University, USA	2010-2015
Naltrexone for Opioid Dependent Released Human Immunodeficiency Virus Positive (HIV+) Criminal Justice Populations	Randomised controlled trial	Effect of depot-naltrexone for opioid- dependent HIV- positive prisoners transitioning to the community	Medication assisted therapy - Depot-naltrexone	HIV-1 RNA level, CD4 count, retention in care, opiate treatment outcome, HIV risk behaviour, rate of reincarceration	Connecticut and Massachusetts, USA	Yale University, USA	2010-2015
Peer-driven Interventions to Seek, Test and Treat Heterosexuals at High Risk for HIV (HHR)	To be determined	Peer-driven interventions to overcome barriers to seek, test, treat HHR	Peer-driven HIV testing and treatment	Efficacy of multi-level peer-driven interventions	New York City, USA	New York University, USA	Planning Phase
HIV, Buprenorphine, and the Criminal Justice System	Randomised controlled trial	Efficacy of buprenorphine in improving ART outcomes for opiate-addicted prisoners transitioning to the community	Medication assisted therapy - Buprenorphine	ART adherence, retention in care, HIV transmission	Washington DC, USA	Yale University, USA	2010-2015
					**GLOBAL**		
START - Strategic Timing of Antiretroviral Treatment	Randomised controlled trial	Effects of early ART initiation	ART initiation at CD4 count >500 cells/mm^3^	Chances of developing AIDS and other illnesses, and drug resistance, quality of life, health care utilisation and cost of care	37 countries	University of Minnesota, Minnesota, USA	2009-2015
Test and Treat to End AIDS (TTEA)	Population-based observational study	Implementation research on test and treat strategy	Large-scale testing and early ART	HIV transmission at population level, long-term costs and mortality	Multi-site (3 or more countries)	TTEA, Lundy Foundation, USA	2011 onwards
The Reducing Early Mortality and Early Morbidity by Empiric Tuberculosis Treatment Regimens (REMEMBER) study (ACTG 5274)	Randomised controlled trial	Impact of ART and empiric treatment for TB	Empiric TB treatment or local standard of care TB treatment	AIDS progression; virologic and CD4+ cell response; HIV and TB drug resistance; safety and tolerability of and adherence to HIV and TB drugs; cost-effectiveness of the two strategies	All of the 18 international ACTG sites are eligible to participate. (Haiti, Peru, Brazil, South Africa, Zimbabwe, Zambia, Tanzania, Kenya, Uganda, Botswana, Malawi, India and Thailand).	Multiple, to be determined	2011-2014 (96 weeks+ 9 months)

## APPENDIX

Alex Welte
  South African Department of Science and Technology/National Research Foundation Centre of Excellence in Epidemiological Modelling and Analysis (SACEMA)
  University of Stellenbosch, Stellenbosch, South Africa,
  alexwelte@sun.ac.za
Alison End
  Country Manager, CHAI Swaziland
  Mbabane, Swaziland
  aend@clintonhealthaccess.org
Ann Duerr
  Fred Hutchinson Cancer Research Center
  HIV Vaccine Trials Network
  Seattle, WA, USA
  aduerr@hvtn.org
Ann Kurth
  Professor, New York University College of Nursing (NYUCN)
  Executive Director, NYUCN Global
  New York, NY, USA
  akurth@nyu.edu
Carlos del Rio
  AITRP Program Director
  Hubert Department of Global Health, Rollins School of Public Health
  Atlanta, Georgia, USA
  cdelrio@emory.edu
Christopher Gordon
  Chief, Secondary HIV Prevention and Translational Research Branch
  Associate Director for Prevention
  National Institute of Mental Health
  Rockville, MD, USA
  cgordon1@mail.nih.gov
Connie L Celum
  Professor of Global Health and Medicine, Adjunct Professor of Epidemiology
  Director, International Clinical Research Center
  Department of Global Health, University of Washington
  Seattle, WA, USA
  ccelum@u.washington.edu
Curt Beckwith
  Associate Professor of Medicine
  Alpert Medical School of Brown University/The Miriam Hospital
  Providence, RI
  CBeckwith@Lifespan.org
David W. Seal
  Professor
  Department of Psychiatry and Behavioral Medicine, Medical College of Wisconsin
  Milwaukee, WI, USA
  dseal@mcw.edu
David Wohl
  Associate Professor, Division of Infectious Diseases at the University of North Carolina
  Site Leader, AIDS Clinical Trials Unit
  The University of North Carolina
  Chapel Hill, NC, USA
  wohl@med.unc.edu
Diane V. Havlir
  University of California
  San Francisco, USA
  dhavlir@php.ucsf.edu
Edward Mills
  Associate Professor and Canada Research Chair, Global Health
  Faculty of Health Sciences
  University of Ottawa, Ottawa, Ontario, Canada
  edward.mills@uottawa.ca
Faye Malitz
  Director, Division of Science and Policy
  Health Resources and Services Administration (HRSA)
  Rockville, MD, USA
  Faye.Malitz@hrsa.hhs.gov
Gary Marks
  Division of HIV/AIDS Prevention - Surveillance and Epidemiology
  National Center for HIV, STD, and TB Prevention
  Atlanta, GA, USA
  gdm8@cdc.gov
Georgina Caswell
  Programme Officer
  Global Network of People Living with HIV (GNP+)
  Amsterdam, The Netherlands
  gcaswell@gnpplus.net
Gilles Van Cutsem
  Medical Coordinator, Medecins Sans Frontieres (MSF)
  South Africa
  gilles.van.cutsem@brussels.msf.org
Grant Colfax
  Director of HIV Prevention and Research
  San Francisco Department of Public Health
  San Francisco, CA, USA
  Grant.Colfax@sfdph.org
Irene Kuo
  Assistant Research Professor
  George Washington University
  School of Public Health and Health Services
  Department of Epidemiology and Biostatistics
  Washington, DC, USA
  sphirk@gwumc.edu
Izukanji Sikazwe
  Internal Medicine, Infectious Diseases
  Medical Technical Advisor National ARV Program
  Ministry of Health
  Lusaka, Zambia, Zambia
  isikazwe@umsom-ihvzambia.org
James D. Neaton
  Professor, Division of Biostatistics
  University of Minnesota School of Public Health
  Minneapolis, MN, USA
  neato001@umn.edu
Jonathan Mermin, Director
  Division of HIV/AIDS Prevention
  CDC; Atlanta, GA, USA
  jhm7@cdc.gov
Joseph Amon
  Director, Health and Human Rights Division
  Human Rights Watch
  New York, New York, USA
  amonj@hrw.org
Josiah Rich
  Director of the Center for Prisoner Health and Human Rights at the Miriam Hospital
  Professor of Medicine and Community Health, Brown Medical School
  Providence, RI, USA
  JRich@lifespan.org
Kevin De Cock
  Director, Center for Global Health
  Centers for Disease Control and Prevention
  Atlanta, Georgia, USA
  kmd2@cdc.gov
Lawrence J. Ouellet
  Research Professor
  Community Outreach Intervention Projects
  Division of Epidemiology and Biostatistics
  School of Public Health
  University of Illinois at Chicago
  Chicago, IL, USA
  ljo@uic.edu
Lisa Metsch
  Professor, Dept. of Epidemiology and Public Health
  University of Miami Miller School of Medicine
  Miami, Florida, USA
  lmetsch@med.miami.edu
Louise van Deth
  Director, STOP AIDS NOW!
  Amsterdam, The Netherlands
  lvandeth@stopaidsnow.nl
Lytt I. Gardner
  Epidemiologist
  Division of HIV/AIDS, Epi Branch
  Centers for Disease Control
  Atlanta GA, USA
  lig0@cdc.gov
Maria Mercedes Santoro
  University of Rome Tor Vergata, Department of Experimental Medicine and Biochemical Sciences
  Rome, Italy
  santormaria@gmail.com
Marie-Louise Newell
  Director, Africa Centre for Health and Population Studies, University of KwaZulu-Natal
  Professor of Health and Population Studies, University of KwaZulu-Natal
  Professor of Paediatric Epidemiology, Institute of Child Health, University College London
  mnewell@africacentre.ac.za
Mark Harrington
  Executive Director
  Treatment Action Group
  New York City, NY, USA
  Mark.Harrington@treatmentactiongroup.org
Michael S. Gordon
  Research Scientist
  Friends Research Institute Inc.
  Baltimore, MD, USA
  mgordon@friendsresearch.org
Moupali Das
  Director of Research, HIV Prevention Section
  San Francisco Department of Public Health
  Assistant Clinical Professor of Medicine, HIV/AIDS and Infectious Diseases, San Francisco General Hospital, UCSF
  San Francisco, California, USA
  dasm@php.UCSF.edu
Nathan Ford
  Medical co-ordinator, Medecins Sans Frontieres
  Geneva, Switzerland
  nathan.ford@msf.org
Navneet Garg
  Chief Development Officer
  Vestergaard Frandsen
  Lausanne Switzerland
  ng@vestergaard-frandsen.com
Olivia**Keiser
  University of Bern
  Institute of Social and Preventive Medicine
  Finkenhubelweg, Bern
  Switzerland
  okeiser@ispm.unibe.ch
Peter Cherutich
  Head of HIV Prevention
  National AIDS/STD Control Programme (NASCOP), Ministry of Health
  Nairobi, Kenya
  pcheru2000@yahoo.com
Rachel M. Cohen
  Regional Executive Director
  Drugs for Neglected Diseases initiative (DNDi), North America
  New York, NY, USA
  rcohen@dndi.org
Redonna Chandler
  Branch Chief
  Division of Epidemiology, Services and Prevention Research (DESPR)
  National Institute on Drug Abuse, National Institutes on Health
  Bethesda, MD, USA
  rc274k@nih.gov
Richard Lester
  Assistant Clinical Professor
  University of British Columbia, Dept. of Medicine, Division of Infectious Diseases
  Vancouver, British Columbia, Canada
  rlester.id@gmail.com, richard.lester@bccdc.ca
Robert Sheneberger
  Assistant Professor, Institute of Human Virology
  University of Maryland School of Medicine
  Baltimore, MD, USA
  rsheneberger@ihv.umaryland.edu
Roger Tatoud
  Senior Programme Manager
  International HIV Clinical Trials Research Mgmt Office
  Imperial College
  London, UK
  r.tatoud@imperial.ac.uk
Ronald Scott Braithwaite
  Associate Professor
  Department of Medicine, New York University Langone Medical Center
  New York, NY, USA
  Scott.Braithwaite@nyumc.org
Sandra Springer
  Assistant Professor of Medicine
  Yale School of Medicine
  Department of Internal Medicine
  Section of Infectious Disease
  Yale AIDS Program
  New Haven, CT, USA
  sandra.springer@yale.edu
Sarah J. Fidler
  CSL in Communicable Diseases, Department of Medicine
  Department of Medicine
  Imperial College
  London, UK
  s.fidler@imperial.ac.uk
Sharonann Lynch
  HIV/AIDS Policy Advisor
  Access to Essential Medicines Campaign
  Médecins Sans Frontières (MSF)
  Geneva, Switzerland
  sharonann.lynch@msf.org
Shruti Mehta
  Department of Epidemiology
  615 N Wolfe Street, E6537
  Baltimore, MD 21205
  443.287.3837 (phone)
  410.955.1383 (fax)
  shmehta@jhsph.edu
Till Bärnighausen
  Senior Epidemiologist, Africa Centre for Health and Population Studies, University of KwaZulu-NatalAssistant Professor of Global Health, Department of Global Health and Population, Harvard School of Public Health
  Timothy D. Mastro
  Vice President
  FHI 360
  Durham, North Carolina, USA
  tmastro@fhi360.org
Veronica Miller
  Associate Research Professor
  The George Washington University, School of Public Health and Health Services
  Washington DC, USA
  vmiller@gwu.edu
Victor Dukay
  President
  Lundy Foundation
  300 West 11th Ave., Suite 15B
  Denver, Colorado 80204-3690, USA
  vic@lundyfoundation.org
Viviane Dias Lima
  Senior Statistician
  Epidemiology and Population Health, Clinical Programs, Drug Treatment Program
  British Columbia Centre* for* Excellence *in* HIV/AIDS
  Vancouver, BC, Canada
  vlima@cfenet.ubc.ca
Vu Minh Quan
  Assistant Professor, Department of Epidemiology,
  Johns Hopkins University Bloomberg School of Public Health
  Baltimore, MD, USA
  vquan@jhsph.edu
Wafaa El-Sadr
  Professor
  Columbia University Mailman School of Public Health
  New York, NY, USA
  wme1@columbia.edu
Warren O’Briain
  Executive Director
  Communicable Disease, Harm Reduction and Mental Health Promotion,
  BC Ministry of Health
  Warren.obriain@gov.bc.ca
William E. Cunningham
  Faculty Associate, UCLA Center for Health Policy Research
  Professor, UCLA School of Medicine
  Los Angeles, California, USA
  wcunningham@mednet.ucla.edu
Xavier Anglaret
  Senior Researcher
  Université Bordeaux
  Bordeaux, France
  xavier.anglaret@isped.u-bordeaux2.fr
Yvette Fleming
  Senior Advisor, Programme Manager MaxART in Swaziland
  STOP AIDS NOW!Keizersgracht 390, 1016 GB Amsterdam yfleming@stopaidsnow.nl
  www.stopaidsnow.org
Zunyou Wu
  Director, National Center for AIDS/STD Control and Prevention
  Chinese Center for Disease Control and Prevention
  Changping District, Beijing, China
  wuzunyou@chinaaids.cn

## ABBREVIATIONS

ACTG: = AIDS Clinical Trials Group
AIDS: = Acquired Immune Deficiency Syndrome
ANRS: = Agence Nationale de Recherche sur le SIDA et les hepatites virales (National Agency for Research on AIDS and Viral Hepatitis, France)
ART: = Antiretroviral Therapy
BCCfE: = British Columbia Centre for Excellence in HIV/AIDS
CDC: = (United States) Centers for Disease Control and Prevention
CHAI: = Clinton Health Access Initiative
CHF: = Cooperative Housing Foundation
CHIP: = Copenhagen HIV Programme
CJS: = Criminal Justice System
CONNECT: = Clinic Oriented New Patient Navigation to Encourage Connection to Treatment (Model)
CTN: = Clinical Trials Network
DISCO: = Discordant Couples (Cohort Team)
DC: = District of Columbia
EPIC: = Enhance Prevention in Couples
FI: = Financial Incentive
GAP: = Global AIDS Program
HAART: = Highly Active Antiretroviral Therapy
HBCT: = Home-Based Counselling and Testing
HCV: = Hepatitis C Virus
HHR: = Heterosexuals at High Risk for HIV
HIV: = Human Immunodeficiency Virus
HIV+: = HIV Positive
HOPE: = Hospital Visit as an Opportunity for Prevention and Engagement for HIV- infected Drug Users
HPTN: = HIV Prevention Trials Network
ICAP: = International Center for AIDS Care and Treatment Programs
ICT: = Information and Communication Tools
IDU: = Injection Drug Users
INSIGHT: = International Network for Strategic Initiatives in Global HIV Trials
IPD: = Integrated Prevention Demonstration (Campaign)
iTLCT: = International HIV Testing and Linkage to Care and Treatment (Study)
MAT: = Medication-Assisted Therapy
MaxART: = Maximizing ART
MDGs: = Millennium Development Goals
MDR: = Multi-Drug Resistant
MSF: = Medecins sans Frontieres (Doctors without Borders)
MSM: = Men Who Have Sex with Men
NCAIDS: = National Center for AIDS/STD Control and Prevention
NIAID: = United States National Institute of Allergy and Infectious Diseases
NIH: = United States National Institutes of Health
PARTNER: = Partners of People on ART: a New Evaluation of the Risks (Study)
PEPFAR: = United States President’s Emergency Plan for AIDS Relief
PopART: = Population Effects of Anti-Retroviral Therapy (Study)
RCT: = Randomised Controlled Trial
REMEMBER: = Reducing Early Mortality and Early Morbidity by Empiric Tuberculosis Treatment Regimens (Study)
SEARCH: = Sustainable East Africa Research for Community Health
SHIMS: = Swaziland HIV Incidence Measurement Survey
SOC: = Standard of Care
START: = Strategic Timing of Antiretroviral Treatment
STI: = Sexually Transmitted Infection
STOP: = Seek and Treat for Optimal Prevention of HIV/AIDS HIV/AIDS (Initiative)
STT: = Seek, Test, (and) Treat
TASO: = The AIDS Support Organisation (Uganda)
TasP: = Treatment as Prevention
TB: = Tuberculosis
TLC: = Testing and Linkage to Care (or Testing, Linkage, and Care)
TTEA: = Test and Treat to End AIDS
UK: = United Kingdom
UNAIDS: = Joint United Nations Programme on HIV/AIDS
US(A): = United States (of America)
UTT: = Universal Testing and Treatment
VCT: = Voluntary Counselling and Testing
WHO: = World Health Organization

## References

[1] Schwartlander B, Stover J, Hallett T (2011). Towards an improved investment approach for an effective response to HIV/AIDS. Lancet.

[2] (2010). WHO. Towards universal access: Scaling up priority HIV/AIDS interventions in the health sector. WHO Geneva Switzerland.

[3] Fang C, Hsu H, Twu S (2004). Decreased HIV transmission after a policy of providing free access to highly active antiretroviral therapy in Taiwan. J Infect Dis.

[4] Das M, Chu PL, Santos GM (2010). Decreases in community viral load are accompanied by reductions in new HIV infections in San Francisco. PLoS ONE.

[5] Montaner JS, Hogg R, Wood E (2006). The case for expanding access to highly active antiretroviral therapy to curb the growth of the HIV epidemic. Lancet.

[6] Granich RM, Gilks CF, Dye C, DeCock KM, Williams BG (2009). Universal voluntary HIV testing with immediate antiretroviral therapy as a strategy for elimination of HIV transmission: a mathematical model. Lancet.

[7] Quinn TC, Wawer MJ, Sewankambo N (2000). Viral load and heterosexual transmission of human immunodeficiency virus type 1.Rakai Project Study Group. N Engl J Med.

[8] Attia S, Egger M, Muller M, Zwahlen M, Low N (2009). Sexual transmission of HIV according to viral load and antiretroviral therapy: systematic review and meta-analysis. AIDS.

[9] Cohen MSCY, McCauley M, Gamble T (2011). the HPTN 052 Study Team.Prevention of HIV-1 infection with early antiretroviral therapy. N Engl J Med.

[10] Lawn SD, Kranzer K, Wood R (2009). Antiretroviral therapy for control of the HIV-associated tuberculosis epidemic in resource-limited settings. Clin Chest Med.

[11] Williams BG, Granich R, Chauhan LS, Dharmshaktu NS, Dye C (2005). The impact of HIV/AIDS on the control of tuberculosis in India. Proc Natl Acad Sci USA.

[12] UNAIDS HIV Prevention Revolution. U AIDS Geneva, Switzerland 2010. Available at http://www.hivpreventioncommi ssion.com/index.php?q=node/43.

[13] Auvert B, Sobngwi-Tambekou J, Cutler E (2009). Effect of male circumcision on the prevalence of high-risk human papillomavirus in young men: results of a randomized controlled trial conducted in Orange Farm, South Africa. J Infect Dis.

[14] Quinn TC (2007). Circumcision and HIV transmission. Curr Opin Infect Dis.

[15] Mills E, Cooper C, Anema A, Guyatt G (2008). Male circumcision for the prevention of heterosexually acquired HIV infection: a meta-analysis of randomized trials involving 11,050 men. HIV Med.

[16] (2010). WHO, UNAIDS. Progress in male circumcision scale-up: country
implementation and research update. WHO/UNAIDS, Geneva,
Switzerland 2010. Available at: www.malecircumcision.org/docum
ents/MC_country_June2010.pdf.

[17] Shapiro RL, Hughes MD, Ogwu A Antiretroviral regimens in pregnancy and breast-feeding in Botswana. N Engl J Med.

[18] Sturt AS, Dokubo EK, Sint TT Antiretroviral therapy (ART) for treating HIV infection in ART-eligible pregnant women. Cochrane Database Syst Rev.

[19] Safety and effectiveness of antiretroviral drugs during pregnancy delivery and breastfeeding for prevention of mother-to-child transmission of HIV-1: the Kesho Bora Multicentre Collaborative Study rationale, design, and implementation challenges. Contemp Clin Trials.

[20] de Vincenzi I (2011). Triple antiretroviral compared with zidovudine and single-dose nevirapine prophylaxis during pregnancy and breastfeeding for prevention of mother-to-child transmission of HIV-1 (Kesho Bora study): a randomised controlled trial. Lancet Infect Dis.

[21] Connor EM, Sperling RS, Gelber R, Pediatric AIDS Clinical Trials Group Protocol 076 Study Group (1994). Reduction of maternal-infant transmission of human immunodeficiency virus type 1 with zidovudine treatment. N Engl J Med.

[22] McCoy SI, Kangwende RA, Padian NS (2010). Behavior change interventions to prevent HIV infection among women living in low and middle income countries: a systematic review. AIDS Behav.

[23] Padian NS, McCoy SI, Balkus JE, Wasserheit JN (2010). Weighing the gold in the gold standard: challenges in HIV prevention research. AIDS.

[24] Kurth AE, Celum C, Baeten JM, Vermund SH, Wasserheit JN (2011). Combination HIV prevention: significance, challenges, and opportunities. Curr HIV/AIDS Rep.

[25] Galea JT, Kinsler JJ, Salazar X (2011). Acceptability of pre-exposure prophylaxis as an HIV prevention strategy: barriers and facilitators to pre-exposure prophylaxis uptake among at-risk Peruvian populations. Int J STD AIDS.

[26] Padian NS, McCoy SI, Manian S, Wilson D, Schwartlander B, Bertozzi S (2011). Evaluation of Large-Scale Combination HIV Prevention Programs: Essential Issues. J Acquir Immune Defic Syndr.

[27] Springer SA, Chen S, Altice FL (2010). Improved HIV and Substance Abuse Treatment Outcomes for Released HIV-Infected Prisoners: The Impact of Buprenorphine Treatment. J Urban Health.

[28] Batkis M (2010). Integrated opioid use disorder and HIV treatment: rationale, clinical guidelines for addiction treatment, and review of interactions of antiretroviral agents and opioid agonist therapies. AIDS Patient Care STDs.

[29] Springer SA (2010). Commentary on Larney (2010): a call to action-opioid substitution therapy as a conduit to routine care and primary prevention of HIV transmission among opioid-dependent prisoners. Addiction.

[30] Larney S (2010). Does opioid substitution treatment in prisons reduce injecting-related HIV risk behaviours? A systematic review. Addiction.

[31] (2010). WHO. Priority interventions HIV/AIDS prevention, treatment and
care in the health sector (2010 version). WHO, Geneva,
Switzerland.

[32] (2009). WHO. Consultation on Antiretroviral Treatment for Prevention of
HIV Transmission - Meeting Report. 2-4 November 2009. WHO,
Geneva, Switzerland.

[33] International Workshop on Treatment as Prevention, 2011,
Vancouver, British Columbia, Canada. Available at: http://www.c fenet.ubc.ca/node/5534.

[34] Discordant Couples (DISCO) Cohort Team. Available at: http:// www.captnetwork.org/disco.html.

[35] An HIV Prevention Program for Mochudi, Botswana. Communitybased
study. Harvard School of Public Health, Boston, USA.
Available at: http://projectreporter.nih.gov/project_info_descriptio
n.cfm?projectnumber=1R01AI083036-01, http://www.labome.org
/grant/r01/ai/an/hiv/an-hiv-prevention-program-for-mochudi--
botswana-7680502.html.

[36] PopART (Population Effects of ART) trial (HPTN071).
Randomized controlled trial. London School of Hygiene and
Tropical Medicine and Imperial College, London, UK. Available
at: http://www1.imperial.ac.uk/departmentofmedicine/divisions/ infectiousdiseases/infectious_diseases/hiv_trials/hiv_prevention_technologies/popart/.

[37] Tanser F, Hosegood V, Bärnighausen T (2008). Cohort Profile: Africa Centre Demographic Information System (ACDIS) and population-based HIV survey. Int J Epidemiol.

[38] Bärnighausen T, Tanser F, Newell ML (2009). Lack of a decline in HIV incidence in a rural community with high HIV prevalence in South Africa, 2003-2007. AIDS Res Hum Retroviruses.

[39] Health, Economic and Education Outcomes of Universal ART. Community randomized control trial. University of California, San Francisco, USA.

[40] Stop AIDS Now! Dutch Post Lottery Dream Fund 2011. Available
at: http://www.novamedia.nl/web/Press/2011-February.-2.5-millio n-Dutch-raise-270-million-for-charity.htm.

[41] Early Antiretroviral Treatment and/or Early Isoniazid Prophylaxis
against Tuberculosis in HIV-infected Adults. Randomized control
trial (TEMPRANO). Université Bordeaux, France. Available at: http://www.clinicaltrial.gov/ct2/show/NCT00495651?term=ART+HIV&rank=78.

[42] Immunology and Outcomes after HAART in HIV/TB co-infection. Cohort study. University of Pennsylvania. Available at: http://projectreporter.nih.gov/project_info_description.cfm?aid=8016040&icde=8287787.

[43] Enhance Prevention in Couples (EPIC). Randomized controlled trial. Columbia University Health Sciences Available at: http://projectreporter.nih.gov/project_info_description.cfm?aid=7860625&icde=8287802.

[44] (2011). Multi-component targeted HIV Prevention for Sub-Saharan Africa: PreventionRX. Randomized controlled trial. University of Washington. Available at: http://projectreporter.nih.gov/project_ info_description.cfm?aid=8077722&icde=8223602.

[45] Interventions to Decrease HIV Infectiousness in Uganda and South
Africa. Community randomized trial. University of Washington.
Available at: http://projectreporter.nih.gov/project_info_description.cfm?aid=8044884&icde=8223602.

[46] HIV/HAART and Pregnancy/Contraception in Rakai, Uganda.
John Hopkins University, Baltimore, USA. Available at: http://projectreporter.nih.gov/project_info_description.cfm?aid=8048171&icde=8223602.

[47] Impact of HIV, ART and TB Genotype on Survival in MDR TB.
Albert Einstein College of Medicine. Available at: http://projectreporter.nih.gov/project_info_description.cfm?aid=8012916&icde=8206866.

[48] Lugada E, Millar D, Haskew J (2010). Rapid implementation of an integrated large-scale HIV counseling and testing, malaria, and diarrhea prevention campaign in rural Kenya. PLoS ONE.

[49] Gender-specific Combination HIV Prevention for Youth in
High/burden Settings (MP3-Youth). New York University.
Available at: http://projectreporter.nih.gov/project_info_descript ion.cfm?aid=8115703&icde=8564348.

[50] Swaziland HIV Incidence Measurement Survey (SHIMS).
Swaziland Ministry of Health. Available at: http://cumc. columbia.edu/dept/icap/wherewework/swaziland/index.html.

[51] Dunkle KL, Stephenson R, Karita E (2008). New heterosexually transmitted HIV infections in married or cohabiting couples in urban Zambia and Rwanda: an analysis of survey and clinical data. Lancet.

[52] HIV Prevention Trials Network's HPTN-070 IHT, Linkage to Care
and Treatment (iTLCT). Available at: http://www.hptn.org/ prevention_science/art.asp.

[53] Seek, Test, Treat Strategies for Vietnamese Drug Users: A Random
Controlled Trial. John Hopkins University, Baltimore, USA.
Available at: http://projectreporter.nih.gov/project_info_description.cfm?aid=8056406&icde=8228568.

[54] Treatment as Prevention Strategy in China. National Center for
AIDS/STD Control and Prevention (NCAIDS), China CDC and
British Columbia Centre for Excellence in HIV/AIDS. Available
at: http://www.cfenet.ubc.ca/news/releases/chinaimplements-bc-cfe%E2%80%99s-treatment-prevention-strategy-country%E2%80%99s-national-hivaids-pol.

[55] Wu Z (2011). HIV prevention studies among high risk groups in China. A
presentation at summary meeting of mega research projects on
AIDS and Hepatitis major infectious diseases in China. Beijing.

[56] Partners of people on ART: a New Evaluation of the Risks
(PARTNER study). Observational study. Copenhagen HIV
Programme (CHIP), Denmark and Royal Free and UC Medical
School, UK. Available at: http://www.partnerstudy.eu/.

[57] Milloy MJ, Buxton J, Wood E, Li K, Montaner JS, Kerr T (2009). Elevated HIV risk behaviour among recently incarcerated injection drug users in a Canadian setting: a longitudinal analysis. BMC Public Health.

[58] Wood E, Kerr T, Marshall BD (2009). Longitudinal community plasma HIV-1 RNA concentrations and incidence of HIV-1 among injecting drug users: prospective cohort study. Br Med J.

[59] Krusi A, Milloy MJ, Kerr T (2010). Ongoing drug use and outcomes from highly active antiretroviral therapy among injection drug users in a Canadian setting. Antivir Ther.

[60] Loutfy MR, Genebat M, Moore D (2010). A CD4+ cell count <200 cells per cubic millimeter at 2 years after initiation of combination antiretroviral therapy is associated with increased mortality in HIV-infected individuals with viral suppression. J Acquir Immune Defic Syndr.

[61] Lima VD, Harrigan R, Bangsberg DR (2009). The combined effect of modern highly active antiretroviral therapy regimens and adherence on mortality over time. J Acquir Immune Defic Syndr.

[62] Kitahata MM, Gange SJ, Abraham AG (2009). Effect of early versus deferred antiretroviral therapy for HIV on survival. N Engl J Med.

[63] Anema A, Au-Yeung CG, Joffres M (2011). Estimating the impact of expanded access to antiretroviral therapy on maternal, paternal and double orphans in sub-Saharan Africa, 2009-2020. AIDS Res Ther.

[64] Lima VD, Hogg RS, Montaner JS (2010). Expanding HAART treatment to all currently eligible individuals under the 2008 IAS-USA Guidelines in British Columbia, Canada. PLoS ONE.

[65] Montaner JS, Lima VD, Barrios R (2010). Association of highly active antiretroviral therapy coverage, population viral load, and yearly new HIV diagnoses in British Columbia Canada a population-based study. Lancet.

[66] Johnston KM, Levy AR, Lima VD (2010). Expanding access to HAART: a cost-effective approach for treating and preventing HIV. AIDS.

[67] HAART Optimism, Drug Use and Risky Sexual Behaviour among
MSM in British Columbia. Simon Fraser University, USA.
Available at: http://projectreporter.nih.gov/project_info_descript ion.cfm?aid=8138977&icde=8228568.

[68] Effect of HAART Expansion on Community Levels of HIV Viral
Load and HIV Risk Behaviours among MSM in British Columbia.
Simon Fraser University, USA. Available at: http://www.fhs.sfu.ca/ research/activeprojects/effect-of-haart-expansion-on-community-levels-of.

[69] Das M, Chu PL, Santos GM (2010). Decreases in community viral load are accompanied by reductions in new HIV infections in San Francisco. PLoS ONE.

[70] Project HOPE: Hospital Visit as Opportunity for Prevention and
Engagement for HIV-Infected Drug Users (CTN-0049). Available
at: http://www.nida.nih.gov/ctn/ProtocolSelected.php?id=%2069.

[71] TLC+: A Study to Evaluate the Feasibility of an Enhanced Test,
Link to Care, Plus Treat Approach for HIV Prevention in the
United States. Community-based study. Columbia University and
Center for Disease Control and Prevention, USA. Available at: http://www.hptn.org/research_studies/hptn065.asp.

[72] A Randomized Controlled Trial and Cohort Study of HIV Testing
and Linkage to Care. Friends Research Institute, USA. Available
at: http://www.friendsresearch.org/spotlight_on_research.htm, http://projectreporter.nih.gov/project_info_description.cfm?aid=8055730&icde=8206866.

[73] Effectiveness of Peer Navigation to Link Released HIV+ Jail
Inmates to HIV Care. Randomized controlled trial. University of
California, Los Angeles, USA. Available at: http://www.labo
me.org/grant/r01/da/effectiveness/of/effectiveness-of-peernavigation-
to-link-released-hiv--jail-inmates-to-hiv-care-
8056358.html, http://projectreporter.nih.gov/project_info_description.cfm?aid=80
56358&icde=8223602.

[74] Improving Linkage to HIV Care Following Release from
Incarceration. Miriam Hospital, USA. Available at: http://www.
labome.org/grant/r01/da/improving/linkage/improving-linkage-tohiv-
care-following-release-from-incarceration-8056374.html, http://projectreporter.nih.gov/project_info_description.cfm?aid=80
56374&icde=8262670.

[75] Randomized Control Trial of an augmented test, treat, link, &
retain model for North Carolina and Texas Prisoners. University of
North Carolina, USA. Available at: http://projectreporter.nih.gov/ project_info_description.cfm?aid=8057688&icde=8228568.

[76] An Integrated Jail-Prison-Community Model for
Illinois. Community-based study. University of Illinois, Chicago,
USA. Available at: http://projectreporter.nih.gov/project_info_ description.cfm?aid=8057664&icde=8262753, http://www.experts.scival.com/uic/grantDetail.asp?t=ep1&id=9192437&o_id=1&.

[77] Seek, Test, and Treat Strategies. Community-based study. Center
for AIDS Intervention Research, Medical College of Wisconsin,
USA. Available at: http://www.mcw.edu/cair/CurrentResearchPro jects.htm, http://projectreporter.nih.gov/project_info_description.cfm?aid=8055746&icde=8262764.

[78] CARE Corrections: Technology for Jail HIV/HCV Testing,
Linkage, and Care (TLC). Randomized controlled trial. Miriam
Hospital, USA. Available at: http://projectreporter.nih.gov/project_ info_description.cfm?aid=8054015&icde=8222389, http://www.gwumc.edu/sphhs/institutescenters/gwuhivaids/about/faculty/kuo.cfm.

[79] Finding, Testing and Treating High-risk Probationers and Parolees
with HIV. Randomized controlled trial. Research Triangle Institute,
North Carolina, USA. Available at: http://projectreporter. nih.gov/project_info_description.cfm?aid=8054126&icde=8262764.

[80] START Together: HIV Testing and Treatment in and after Jail.
Randomized controlled trial. National Development and Research
Institutes, New York City, USA. Available at: http://project reporter.nih.gov/project_info_details.cfm?aid=8055789&icde=8262814.

[81] START - Strategic timing of Antiretroviral treatment. University of
Minnesota - Clinical and Translational Science Institute, USA.
Available at: http://insight.ccbr.umn.edu/start/.

[82] Test and treat to end AIDS (TTEA). TTEA and Lundy Foundation,
USA. Available at: http://ttea.info/.

[83] Treatment as Prevention (TasP). Community randomized control
trial. University of Bordeaux, Hôpitaux Universitaires de Genève
and Africa Centre for Health and Population Studies. Available at: http://www.africacentre.ac.za/Default.aspx?tabid=439.

[84] WHO Guidelines Review Committee (GRC). Available at: http://www.who.int/kms/guidelines_review_committee/en/index.html.

[85] Guyatt GH, Oxman AD, Kunz R (2008). Going from evidence to recommendations. Br Med J.

[86] Lee DH, Vielemeyer O (2011). Analysis of overall level of evidence behind Infectious Diseases Society of America practice guidelines. Arch Intern Med.

[87] Jensen PA, Lambert LA, Iademarco MF, Ridzon R (2005). Guidelines for preventing the transmission of Mycobacterium tuberculosis in health-care settings, 2005. MMWR Recomm Rep.

[88] WHO. WHO Policy on TB Infection Control in Health-Care
Facilities, Congregate settings and Households. WHO, Geneva,
Switzerland 2009. Available at: http://whqlibdoc.who.int/publicat ions/2009/9789241598323_eng.pdf.

[89] Fee E, Parry M (2008). Jonathan Mann HIV/AIDS, and human rights. J Public Health Policy.

[90] Jones L, Akugizibwe P, Amon J Costing human rights and
community support interventions as a part of universal access to
HIV treatment and care in a southern African setting. XVIII International AIDS Confeence Vienna, Austria. Abstract TUPE1033.

[91] Human Rights Watch. Human Rights Watch. A Testing Challenge: The Experience of
Lesotho's Universal HIV Counseling and Testing Campaign.
Human Rights Watch, 2008. Available at: http://www.hrw.org/en/ reports/2012/11/18/testingchallenge.

[92] Larsson EC, Thorson A, Pariyo G (2011). Opt-out HIV testing during antenatal care: experiences of pregnant women in rural Uganda. Health Policy Plan.

[93] Angotti N, Dionne KY, Gaydosh L (2010). An offer you can't refuse? Provider-initiated HIV testing in antenatal clinics in rural Malawi. Health Policy Plan.

[94] Cohen JE, Amon JJ (2008). Health and human rights concerns of drug user in detention in Guangxi, China. PLoS Med.

[95] UNAIDS. The China Stigma Index Report. UNAIDS and Partners,
Geneva, Switzerland 2009. Available at: http://data.unaids. org/pub/Report/2009/20091127_stigmaindexsummaryreport_en.pdf.

[96] Burris S, Cameron E (2008). The Case Against Criminalization of HIV Transmission. J Am Med Assoc.

[97] Policy Research and Information Division of the National Center
for AIDS/STD Control and Prevention, China CDC. HIV and
AIDS Related Employment Discrimination in China. International
Labour Organization (ILO) Country Office for China and Mongolia
2011. Available at: http://www.ilo.org/asia/whatwedo/publications/ lang--en/docName--WCMS_150386/index.htm.

